# Transcriptomics and Metabolomics Analyses Reveal Defensive Responses and Flavonoid Biosynthesis of *Dracaena cochinchinensis* (Lour.) S. C. Chen under Wound Stress in Natural Conditions

**DOI:** 10.3390/molecules27144514

**Published:** 2022-07-15

**Authors:** Yang Liu, Shixi Gao, Yuxiu Zhang, Zhonglian Zhang, Qiuling Wang, Yanhong Xu, Jianhe Wei

**Affiliations:** 1 Key Laboratory of Bioactive Substances and Resources Utilization of Chinese Herbal Medicine, Ministry of Education National Engineering Laboratory for Breeding of Endangered Medicinal Materials, Institute of Medicinal Plant Development, Chinese Academy of Medical Sciences and Peking Union Medical College, Beijing 100193, China; 18500325600@163.com (Y.L.); shixigao0426@163.com (S.G.); wql19811025@126.com (Q.W.); 2 Hainan Provincial Key Laboratory of Resources Conservation and Development of Southern Medicine & Key Laboratory of State Administration of Traditional Chinese Medicine for Agarwood Sustainable Utilization, Hainan Branch of the Institute of Medicinal Plant Development, Chinese Academy of Medical Sciences and Peking Union Medical College, Haikou 570311, China; scauzyx@163.com; 3 Yunnan Branch of the Institute of Medicinal Plant Development, Chinese Academy of Medical College and Peking Union Medical College, JingHong 666100, China; zzl0605@163.com

**Keywords:** *D. cochinchinensis*, dragon’s blood, flavonoids, metabolites, plant defense, transcriptome, wound-induced

## Abstract

*Dracaena cochinchinensis* has special defensive reactions against wound stress. Under wound stress, *D. cochinchinensis* generates a resin that is an important medicine known as dragon’s blood. However, the molecular mechanism underlying the defensive reactions is unclear. Metabolomics and transcriptomics analyses were performed on stems of *D. cochinchinensis* at different timepoints from the short term to the long term after wounding. According to the 378 identified compounds, wound-induced secondary metabolic processes exhibited three-phase characteristics: short term (0–5 days), middle term (10 days–3 months), and long term (6–17 months). The wound-induced transcriptome profile exhibited characteristics of four stages: within 24 h, 1–5 days, 10–30 days, and long term. The metabolic regulation in response to wound stress mainly involved the TCA cycle, glycolysis, starch and sucrose metabolism, phenylalanine biosynthesis, and flavonoid biosynthesis, along with some signal transduction pathways, which were all well connected. Flavonoid biosynthesis and modification were the main reactions against wound stress, mainly comprising 109 flavonoid metabolites and 93 wound-induced genes. A group of 21 genes encoding CHS, CHI, DFR, PPO, OMT, LAR, GST, and MYBs were closely related to loureirin B and loureirin C. Wound-induced responses at the metabolome and transcriptome level exhibited phase characteristics. Complex responses containing primary metabolism and flavonoid biosynthesis are involved in the defense mechanism against wound stress in natural conditions, and flavonoid biosynthesis and modification are the main strategies of *D. cochinchinensis* in the long-term responses to wound stress.

## 1. Introduction

Biotic and abiotic stresses frequently cause mechanical damages to plants; hence, plants have evolved various strategies to defend themselves against mechanical damages [1]. While suffering through mechanical damages, plants generate signal molecules, activate the expression of defense-related genes, and produce defensive compounds by adjusting the metabolism process to prevent the spread of the injury and secondary damages [2,3,4]. By adjusting the metabolism processes, plants can cooperate with the surrounding environment, defend themselves, and benefit animals. Flavonoids are one of the largest classes among the plant secondary metabolites and have been proved to be closely related to plant defense [5]. Plants which are rich in flavonoids could be used as nutraceutical and medical agents as they have antioxidative, anti-inflammatory, and antimutagenic effects [6,7]. Artificial damages are sometimes performed to efficiently induce plants to produce such secondary metabolites. Agarwood is a typical wound-induced material obtained from *Aquilaria sinensis* and exhibits high medicinal and cultural values. Under wound stress, *A. sinensis* initiates terpenoid and chromone synthesis to produce agarwood to relieve wound-stress. On the basis of this property, *the whole-tree agarwood-inducing technique has been developed for the mass production of agarwood* [8].

*Dracaena cochinchinensis* (Lour.) S. C. Chen is an evergreen arborescent monocotyledon, which is mainly distributed in Yunnan and Guangxi province in China [9]. *D. cochinchinensis* and other arborescent species in *Dracaena* can generate red resinous materials, mainly in the stems, following either naturally formed cracks or incisions formed by external forces [10]. The resin is named dragon blood and is a traditional medicine worldwide [11]. Dragon’s blood is traditionally applied to cure wounds, relieve pain, prevent bleeding, and deal with various diseases such as diarrhea, dysentery, and ulcers [12,13,14]. The Longxue Tongluo capsule that is made of dragon’s blood is clinically used to treat cardiovascular and cerebrovascular diseases [15]. In China, *D. cochinchinensis* is the official origin of dragon’s blood; however, it has been listed as a protected species. As a monocotyledon, *D. cochinchinensis* grows very slowly into trees; in natural conditions, only perennial trees can accumulate a collectible amount of resin after they are damaged for months [16]. However, many *Dracaena* species, which used to be sources of dragon’s blood, are now listed as threatened species and, hence, are no longer exploitable [17,18]. Because of the overexploitation and low yield of dragon’s blood, resources of dragon’s blood are extremely scarce.

Similar to agarwood, dragon’s blood is also a wound-induced material for the self-protection of *Dracaena* spp. The formation of this resinous material is a positive response of *Dracaena* spp. to external stress and noxious mechanical stimulation. It involves a complicated physiological and ecological process based on its special secondary metabolites. Flavonoids are the main active compounds of dragon’s blood [19,20], and loureirin B and C are its indicator compounds for quality evaluation; some studies have explored the flavonoid biosynthesis pathway in *Dracaena* spp. Zhu et al. identified 20 candidate genes in flavonoid biosynthesis, 27 candidate genes in flavonoid modification, and 68 candidate genes in flavonoid transport in *D. cambodiana* [21]. They also proposed that *bHLH*, *MYB, WD 40* transcriptome factor, and *GST* might be related to flavonoid biosynthesis and transport [22,23]. Zhang et al. identified 20 unigenes in flavonoid synthesis and 56 unigenes in flavonoid modification and transport in *D. cochinchinensis*. They annotated *CHS*, *CHI*, *DFR*, *F3′5′H*, *F3H*, *ANR FLS*, and *ANS* in the flavonoid biosynthesis pathway in *D. cochinchinensis* [24]. Flavonoid biosynthesis among plant species is highly conserved and well investigated. The core structure is derived from phenylalanine and malonyl-CoA, and some of its groups can be hydroxylated, glycosylated, methylated or acetylated to form various of flavonoid compounds [25]. Although these studies have provided valuable information on flavonoid biosynthesis in *Dracaena* spp., the interplay of wound-caused stress and the resin formation process of *D. cochinchinensis*, as well as the correlation of metabolism profile and gene expression profile, still remains unclear.

Considering the high medicinal value and wide applications of dragon’s blood, surprisingly little is known about the metabolism and transcriptome profile changes during the defensive reactions of *D. cochinchinensis* caused by wound stress over time. In the present study, we performed transcriptomics and secondary metabolomics analyses on the wounded stems of *D. cochinchinensis* at different timepoints to (1) reveal the characteristics of wound-induced defensive reactions of *D. cochinchinensis* over time, (2) speculate the interplay of wound stress and flavonoid biosynthesis in *D. cochinchinensis*, and (3) predict the key genes related to the synthesis of bioactive flavonoid compounds of dragon’s blood.

## 2. Results

### 2.1. Identification of Secondary Metabolites and Analysis of Differential Metabolites in D. cochinchinensis Stems at Different Timepoints after Wounding

**Figure 1:**
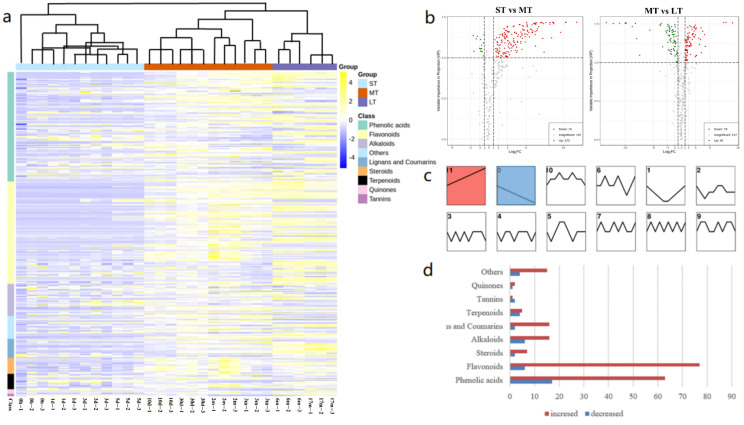
Secondary metabolites of *D. cochinchinensis* stems at different times after wounding: (**a**) heatmap and clustering of metabolic profile of samples at different times after wounding; (**b**) volcano plot of samples in different stages after wounding; (**c**) trend analysis result of samples over time after wounding; (**d**) types of compounds in trend 11 and 0.

A total of 378 compounds were identified from the main stems of *D. cochinchinensis* at different timepoints after wounding. Qualitative and quantitative analysis results of 378 compounds from healthy and wounded stems are listed in Supplementary Materials-Additional File 1. Among these 378 compounds, 128 compounds and 119 compounds were identified as phenolic acids and flavonoids, respectively, followed by alkaloids and lignans/coumarins (37 and 22, respectively) (Figure S1a). At different timepoints after wounding, samples exhibited different secondary metabolites, and the abundance of compounds increased within 30 days after wounding (Figure S1b). PCA showed that the metabolism profiles of samples within the same time group were clustered together, and the distribution of metabolites among different groups according to their similarity was in accordance with the wounding time as expected (Figure S1c). Moreover, the cluster result of the metabolism profile of different timepoints after wounding showed that all samples could be separated into three groups: 0–5 days, 10 days–3 months, and 6–17 months (Figure 1a), indicating the presence of staged secondary metabolism modulation in *D. cochinchinensis* stems under wound stress.

According to the metabolite profiles, the metabolic processes occurring under wound stress exhibited characteristics of three stages (short term: 0–5 days, middle term: 10 days–3 months, and long term, i.e., over 6 months). Differential metabolites (DEMs) were analyzed among the three stages. The volcano plot showed the number of DEMs among different stages (Figure 1b). In the short term, 170 diverse compounds increased, and 15 compounds decreased compared with the short term, indicating a dramatic change in the metabolism level of *D. cochinchinensis* during the preliminary stage under wound stress. From middle to long term, only 82 compounds were upregulated, indicating that some specific metabolic pathways played a dominant role in the later period of wound-induced defensive reactions. The heatmap shows the types of differential compounds at different stages (Figure 1a). Compared with wounded stems, the healthy stems contained a higher amount of steroids, indicating that steroids might not be induced by wound stress, whereas flavonoids and phenolic compounds could be induced at varying degrees under wound stress. In the preliminary stage after wounding, some phenolic acids were mainly enriched in stems. In the middle term, various secondary compounds were increased compared with the short term, indicating a general mobilization of secondary metabolites during defensive responses within this stage. From the middle term to the long term, the overall content of flavonoids increased. In contrast, the relative amount of alkaloids decreased during the later period after wounding. To summarize, similar metabolic patterns appeared at timepoints within the same stages, whereas those at different stages showed specific metabolic patterns. According to the dynamic trend analysis, two groups of metabolites had similar trends with strong significance (*p* < 0.01) after the trees were wounded over time (Figure 1c). A total of 202 compounds in trend 11 continuously increased over time after wounding. These 202 compounds included 76 flavonoids, 63 phenolic acids, and some other types. On the contrary, 44 compounds in trend 0 continuously decreased under wound stress (Figure 1d). It is worth noting that flavonoids showed the most abundance (76 of 202) in the continuously increased compounds, and almost half of these flavonoids (33 of 76) were glycosylated. These 76 flavonoids are listed in Table S2.

### 2.2. General Transcriptomics Analysis and Functional Enrichment of DEGs in D. cochinchinensis Stems at Different Times after Wounding

To reveal the molecular events in *D. cochinchinensis* under wound stress, transcriptomics analysis was performed on stems at different timepoints after wounding. Generally, regulation events at the gene level occur rapidly. Hence, samples at 6 h and 12 h after wounding were used for the analysis. A total of 1,214,276,790 clean reads were acquired (45,377,842 clean reads per sample on average). The percentages of Q20 and GC were 95.45–98.08% and 46.54–49.91%, respectively (Table S3). The percentages of clean reads of all samples totally mapped to the reference genome sequence were 88.77–94.85%, and the uniquely mapped rate was 79.25–89.32%. A total of 34,381 genes were annotated. The Pearson correlation analysis based on the unigene expression showed the reliability of biological repetitions at the same timepoint (Figure S2a). Compared with the healthy stem, wounded stems at different timepoints showed a significant number of DEGs, i.e., between 1171 and 8534 (Figure S2b). The maximum number of DEGs was observed at 24 h, whereas the minimum number of DEGs was observed at 17 m. As shown in Figure S2b, the number of DEGs significantly increased within 24 h after wounding, suggesting complicated wound stress reactions. After 24 h, the number of DEGs showed instability. At 17 m, expression of most genes returned to normal, and only a few DEGs remained upregulated. According to the cluster analysis based on the DEGs at different timepoints, changes at the transcriptome level triggered by wound stress exhibited characteristics of four stages: 0–24 h after wounding, 1–5 days, 10–30 days, and long term (17 months). Overall, the transcriptome sequencing data indicated that mechanical damage could induce obvious changes in the transcriptome of *D. cochinchinensis* stems. Gene annotation and expression quantity are listed in Supplementary Materials-Additional File 2.

**Figure 2:**
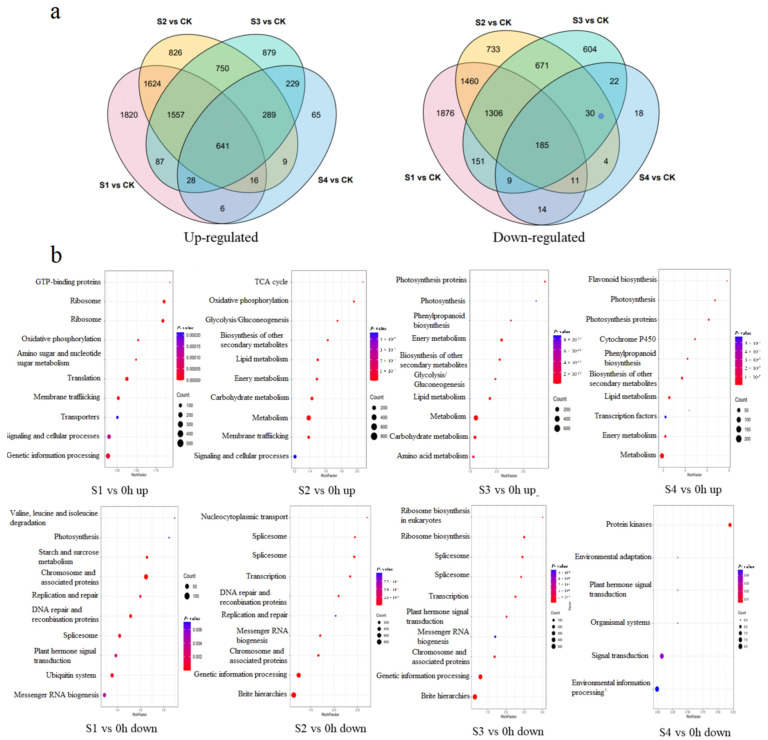
DEG analysis in different stages after wounding: (**a**) Venn diagram of DEGs in different stages; (**b**) KEGG enrichment of DEGs in different stages.

According to the clustering, responses at the genetic level could be roughly divided into four stages: S1, within 24 h; S2, 3–5 days; S3, 10–30 days; S4, 17 months. The upregulated and downregulated DEGs between different stages and 0 h were shown in Figure 2a. A total of 641 genes were upregulated in all four stages, while 185 genes were downregulated in all four stages. S1 had the most abundant DEGs, while S4 had the fewest DEGs. KEGG analysis showed that, in S1, the ribosome, translation, and genetic information processing were the most upregulated pathways, while chromosome and associated proteins, the spliceosome, and the ubiquitin system were the most downregulated pathways. In S2, the TCA cycle, glycolysis/gluconeogenesis, and the biosynthesis of secondary metabolite became upregulated, whereas most genes in protein families, genetic information processing, and the spliceosome were downregulated with a high Rich factor. In S3, glycolysis/gluconeogenesis and biosynthesis of secondary metabolites remained upregulated, and phenypropanoid biosynthesis appeared to be upregulated, whereas the ribosome and spliceosome remained downregulated. In S4, the flavonoid biosynthesis had the highest Rich factor among the upregulated items, while protein kinases had the highest Rich factor among the downregulated items (Figure 2b).

### 2.3. Wound-Activated Molecular Events in Different KEGG Pathways and Their Association

**Figure 3:**
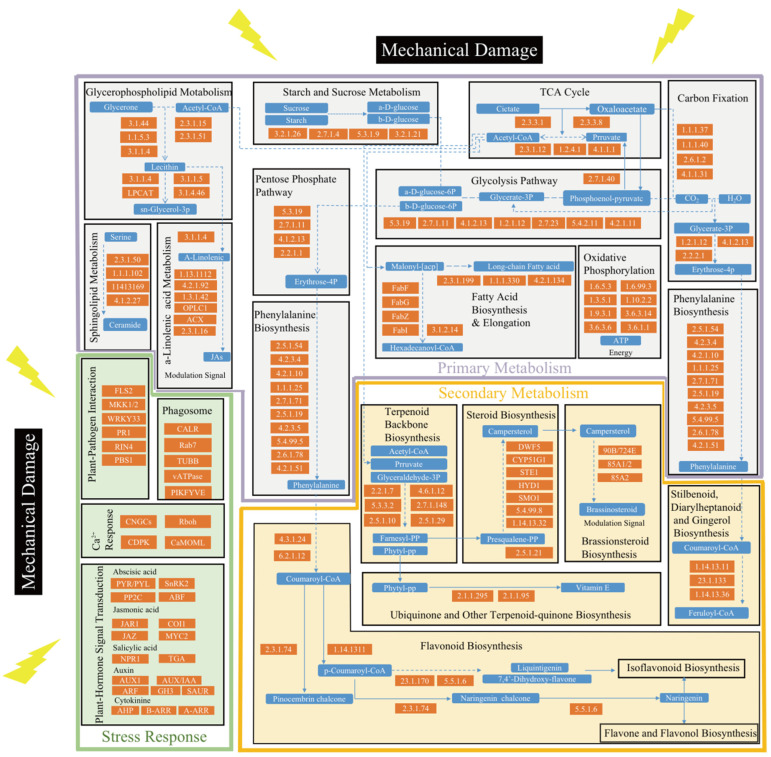
Connected KEGG Pathways of *D. cochinchinensis* under wound stress. The orange frame with numbers indicates upregulated genes; the blue frame indicates key metabolites; the full line indicates one-step reactions; the dotted line indicates reactions with multiple steps and the connections among different pathways.

To identify the most related molecular events to the responses to the mechanical damage and the relationship among these events, genes activated at successive timepoints after wounding from different pathways were screened to tease out the connection among different metabolism pathways (Figure 3). Some specific KEGG pathways could connect molecular events which occurred at different timepoints. In the very early stage after wounding, starch and sucrose metabolism, glycerophospholipid metabolism, and α-linolenic acid metabolism were enriched, indicating that starch and sucrose might be the material basis of the *D. cochinchinensis* defense system, and that jasmonic acid might be an important wounding signal. From 24 h to 10 days, the activated TCA may be primarily responsible for the regulation of primary metabolism under wound stress because most annotated genes in TCA were upregulated during this period. Glycolysis was another important junction station in primary metabolism regulation under wound stress. Pyruvate and acetyl-CoA produced by the TCA and glycolysis could provide the substrate for many other metabolic processes. Pyruvate could be used via the pentose phosphate pathway (mainly enriched from 12 h to 5 days) to generate coumaryl-CoA, which is the collective substrate of many downstream metabolite processes, including flavonoid biosynthesis, as well as stilbenoid diarylheptanoid and gingerol biosynthesis, which were enriched after 3 days. Furthermore, acetyl-CoA could enter terpenoid backbone biosynthesis, which was further connected to the downstream ubiquinone and another terpenoid-quinone biosynthesis (enriched after 3 days) and steroid biosynthesis pathways (enriched from 10 to 30 days). Notably, genes associated with brassinosteroid biosynthesis were nearly all upregulated, which indicated that brassinosteroids might be another important signal of *D. cochinchinensis* under wound stress. In addition, many upregulated genes annotated in oxidative phosphorylation may help ensure the supply of ATP during metabolism regulation in defensive reactions of *D. cochinchinensis*.

Along with the regulation of primary and secondary metabolism, a complex signal transduction network and plant–pathogen interaction were also closely related to the wound-induced reactions in *D. cochinchinensis*. A total of 28 genes in auxin and 10 genes in cytokinin transduction pathways were significantly downregulated from 3 days to 30 days, indicating that the growth processes in *D. cochinchinensis* slowed down under wound stress. Gibberellin receptors (two genes), abscisic acid receptors (eight genes), ethylene receptors (five genes), and salicylic acid receptors (six genes) were upregulated, indicating that gibberellin, abscisic acid, and ethylene may be implicated in the regulation of *D. cochinchinensis* defense reactions induced by mechanical damage. A total of 12 upregulated jasmonic acid receptor genes further supported that jasmonic acid could be the main defensive signal molecule of *D. cochinchinensis*. Genes reacting against bacterial flagellins such as leucine-rich repeat (LRR) receptor-like serine/threonine protein kinase, mitogen-activated protein kinase, and WRKY transcription factor 33 were upregulated from 24 h to 17 months, which implies the presence of a persistent conflict between *D. cochinchinensis* and bacteria after wounding. Moreover, cyclic nucleotide-gated channel, *CDPK*, and *CaMOML* genes were also activated, which showed that wounding signals might pass via Ca^2+^. The information of upregulated genes in Figure 3 is listed in Table S4.

### 2.4. The Combined Analysis and Correlation Network of DEGs and DEMs in D. cochinchinensis Stems at Different Timepoints after Wounding

**Figure 4:**
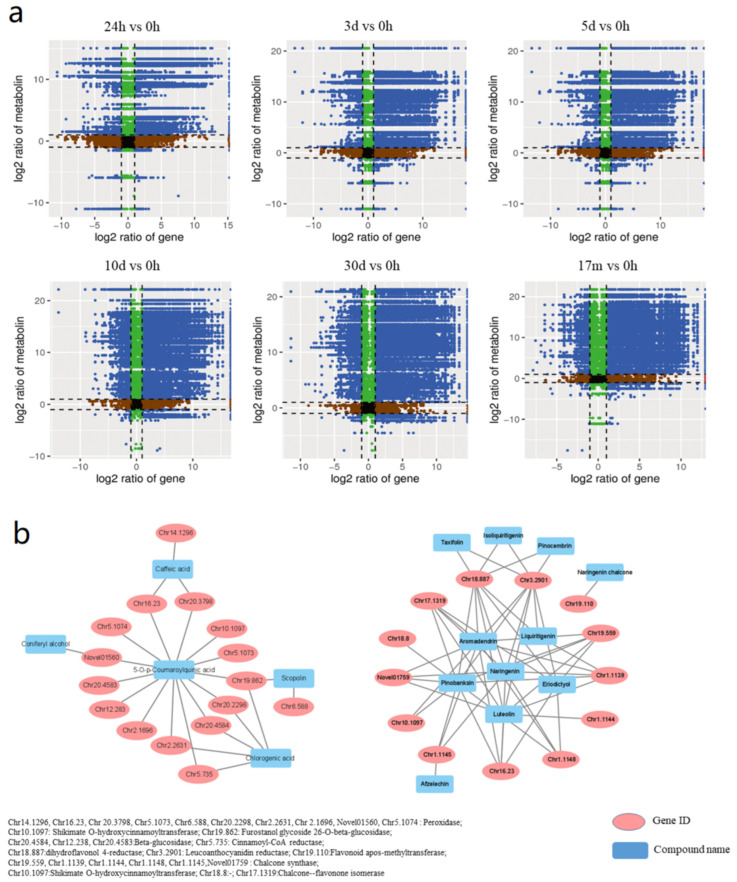
Correlation analysis of DEGs and DEMs: (**a**) nine-quadrant diagram of unigenes and compounds at different wounding timepoints. Unigenes and compounds in quadrants 3 and 7 have accordant tendency; unigenes and compounds in quadrants 1 and 9 have reverse tendency; unigenes and compounds in quadrant 5 are not differentially expressed; unigenes and compounds in quadrants 2, 4, 6, and 8 have no obvious tendency; (**b**) network of DEGs and DEMs in phenylpropanoid biosynthesis and flavonoid biosynthesis pathways.

In general, the difference between transcriptome profiles of the wounded stem became more and more obvious from 24 h to 5 days compared with those of the healthy stem. From 10 days to 17 months, the difference decreased, and, at 17 m, most genes were normally expressed (Figure S2c). The difference in the metabolic level increased from the short term to the middle term, but decreased from the middle term to the long term (Figure 1c). The nine-quadrant diagram (Figure 4a) of DEGs and DEMs at different timepoints showed the presence of a growing tendency of the correlation between DEGs and DEMs from the 24 h to 30 days after wounding, which suggests a close relationship between gene expression and secondary metabolite accumulation during this period after wounding. In the long term after 30 days, some of the correlations still remained. Taken together, core processes were primarily responsible for defense reactions against wound-caused stress from short term to long term in natural conditions.

Combined KEGG was performed on both DEGs and DEMs in samples at different timepoints after wounding for identifying the specific pathways implicated in defensive reactions in *D. cochinchinensis* stems under wound stress. The 15 most enriched pathways at different timepoints according to the *p*-value were selected (Figure S3). In the 1 day sample, DEGs and DEMs were mainly clustered into carbon metabolism, phenylalanine metabolism, stilbenoid, diarylheptanoid, and gingerol biosynthesis, and ubiquinone and other terpenoid-quinone biosynthesis pathways. Except for the metabolic pathways and biosynthesis of secondary metabolites, DEGs and DEMs in phenylpropanoid biosynthesis and flavonoid biosynthesis showed the highest enrichment degree. In addition, phenylpropanoid biosynthesis and flavonoid biosynthesis pathways had the largest number of annotated DEGs and DEMs (Table 1).

The co-expression network of DEGs and DEMs (Pearson correlation coefficient > 0.8 or <−0.8, *p*-value < 0.05) was also mainly enriched in phenylpropanoid pathways and flavonoid biosynthesis (Figure 4b). In the phenylpropanoid pathways network, 16 genes annotated as *cinnamoyl-CoA reductase*, *peroxidase*, *shikimate O-hydroxycinnamoyltransferase*, *furostanol glycoside 26-O-beta-glucosidase*, and *beta-glucosidase* were connected with five compounds, namely, scopolin, 5-*O*-*p*-coumaroylquinic acid, chlorogenic acid, coniferyl alcohol, and caffeic acid. In the flavonoid biosynthesis pathway network, 13 genes annotated as *chalcone synthase*, *shikimate O-hydroxycinnamoyltransferase*, *dihydroflavonol 4-reductase/flavanone 4-reductase*, *leucoanthocyanidin reductase*, *chalcone-flavanone isomerase*, and *flavonoid O-methyltransferase* were connected with 11 compounds, namely, pinobanksin, naringenin, luteolin, aromadendrin, dihydroquercetin, isoliquiritigenin, liquiritigenin, eriodictyol, pinocembrin, naringenin chalcone, and afzelechin. The combined transcriptomics and metabolomics analysis indicated that phenylpropanoid and flavonoid biosynthesis are mainly implicated in the wound-induced defensive reactions of *D. cochinchinensis*.

### 2.5. Flavonoids Biosynthesis Pathway in D. cochinchinensis Stems under Wound Stress

**Figure 5:**
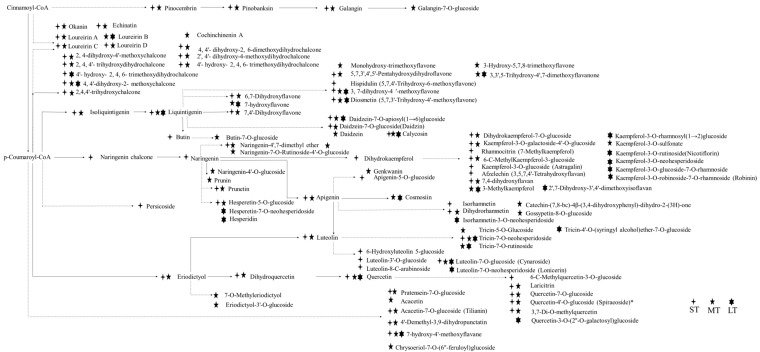
Flavonoid biosynthesis profile at chemical level in *D. cochinchinensis* after wounding. ST, short term; MT, middle term; LT, long term.

The co-expression analysis of DEGs and DEMs led to the conclusion that flavonoid biosynthesis is the main reaction in the defense system of *D. cochinchinensis*, especially in the long term. The flavonoid biosynthesis profile of *D. cochinchinensis* over time after wounding is shown in Figure 5. A total of 119 flavonoid compounds were mapped. Different periods after wounding showed different patterns of flavonoid profiles. Chalcones and flavones were mainly enriched in the short term and early middle term (within 10 days) after wounding, whereas flavonols and isoflavone were mostly enriched after the middle term. Obviously, from the middle term to long term, no new flavonoid compounds were synthesized, and the compounds which already existed increased pretty slowly. In addition, the majority of flavonoids enriched in the long term were flavonoid glycosides. Some compounds such as 4,4′-dihydroxy-2-methoxychalcone, 3,7-dihydroxy-4′-methoxyflavone, liquintigenin, diosmetin, cynaroside, quercetin, and 7-hydroxy-4′-methoxyflavane were persistently synthesized and accumulated from short term to long term under wound stress. Specific differential flavonoid compounds emerging at two neighboring timepoints are listed in Supplementary Materials-Additional File 3, which shows the details of which flavonoid compounds presented at which timepoints and their content changing over time. In the flavonoid profile, loureirin A–D, belonging to the chalcone family, were the main bioactive and index compounds for the quality control of dragon’s blood. Notably, loureirin A, loureirin C, and loureirin D were enriched in the short and middle term, whereas loureirin B was enriched and accumulated in the middle term and long term at a very low speed.

**Figure 6:**
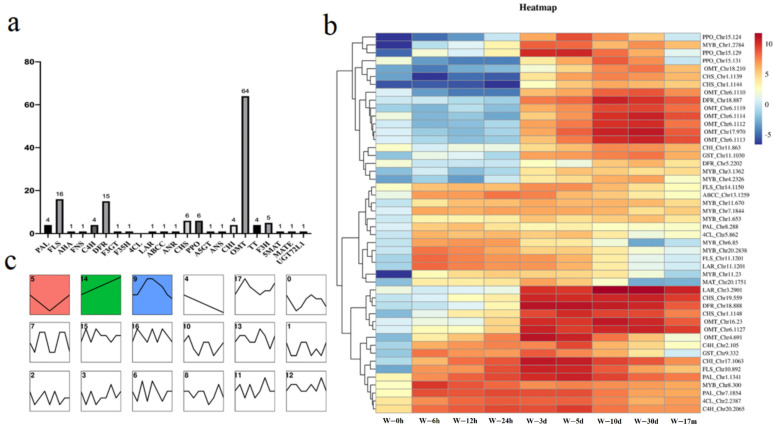
Flavonoid biosynthesis profile at genetic level in *D. cochinchinensis* after wounding: (**a**) types of annotated genes in flavonoid biosynthesis; (**b**) trend analysis result of genes related to flavonoid biosynthesis; (**c**) heatmap and clustering of wound-induced genes related to flavonoid biosynthesis.

We manually inspected the genes in the flavonoid biosynthesis according to the BLAST annotation and used Knowledge-based Identification of Pathway Enzymes (KIPEs) methods [26] to automatically identify the potent genes involved in the flavonoid biosynthesis and transportation in *D. cochinchinensis*. Furthermore, we manually picked out potent *bHLHs*, *WD40*, and *TT* that might regulate the plant flavonoid biosynthesis. Moreover, we applied an MYB annotator program to identify the candidate *MYBs* in *D. cochinchinensis* [27]. As a result, a total of 155 genes annotated in the flavonoid biosynthesis pathways (Figure 6a) and 21 *GST* genes were obtained. A total of 65 *bHLHs*, three *WD40*, and five *TT* were obtained, and 119 genes were annotated to be potent *MYBs* by the MYB annotator. These gene IDs and annotations are listed in Supplementary Materials-Additional File 4. Trend analysis of these genes showed three expression patterns after wounding with high significance (*p* < 0.01) (Figure 6b). A total of 93 genes were found to be upregulated after wounding, and they presented two patterns: continuously increased (60) and increased first before declining (33). In these two patterns, we found that 48 genes might be wound-induced as they were rarely expressed in the healthy stem but highly expressed after wounding. According to the cluster by the expression patterns of the 48 genes over time after wounding, it could be seen that different genes worked in different stages. Some genes (mainly including six *MYBs,* two *PAL* and *4CL*, one *LAR*, and one *FLS*) were activated in the very early stage after wounding (within 24 h) while some genes (mainly including two *FLS* and *PPO*, and one *PAL*, *MYBs*, *CHI*, *CHS*, and *DFR*) were activated within 3–5 days. In addition, it is worth noting that some of these genes (mainly including eight *OMT*, two *DFR* and *CHS*, and one *LAR*) reached their expression peak in the later period (10–30 days); surprisingly, they remained highly expressed even in the long term (over 6 months) after wounding, which might mainly contribute to the accumulation of resin in the long term after wounding (Figure 6c).

**Figure 7:**
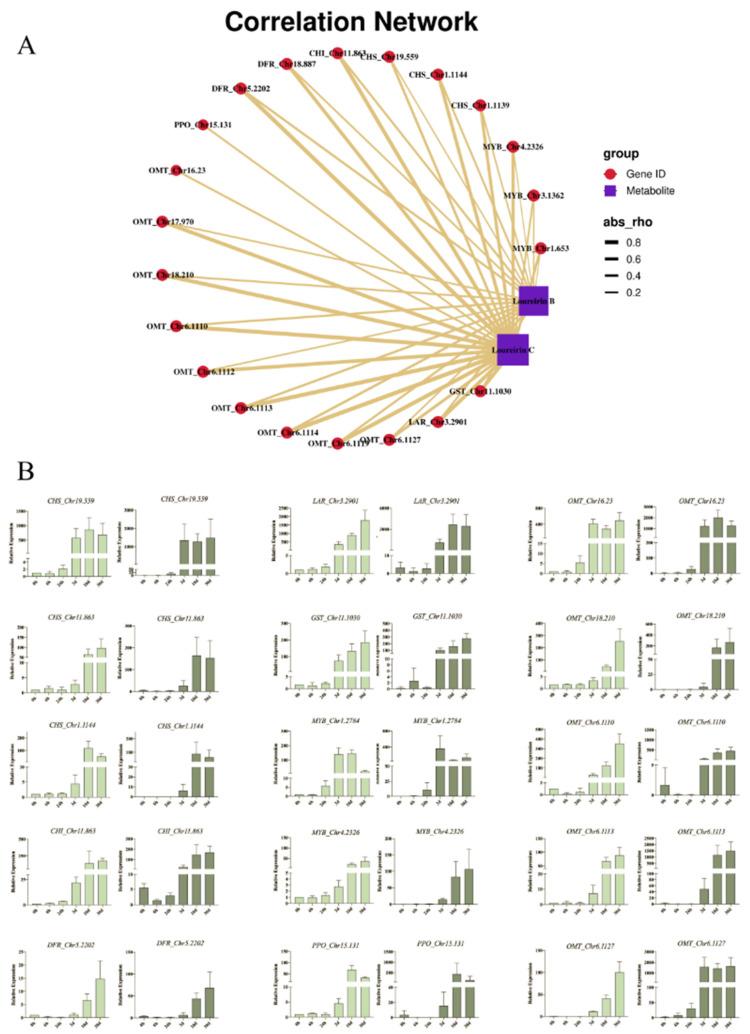
Putative genes related to the biosynthesis of loureirin B and C and their qRT-PCR validation: (**A**) network of putative genes and compounds; (**B**) qRT-PCR results of putative genes.

Correlation network analysis (Pearson correlation coefficient >0.8 or <−0.8) was performed on the basis of wound-induced flavonoid biosynthesis-related genes and loureirin B and loureirin C, the index compounds to evaluate the quality of dragon’s blood (Figure 7A). As a result, nine *OMT*, three *CHS*, two *DFR*, one *CHI*, one *PPO*, one *LAR*, and one *GST,* which were annotated to be flavonoid biosynthesis genes, were closely associated with these two compounds. Furthermore, three *MYBs* might be the possible regulation transcription factors related to these compounds. To further confirm the expression patterns of these genes associated with flavonoid biosynthesis, qRT-PCR was performed on 15 genes, namely, three *CHS*, five *OMT*, two *MYBs*, one *CHI*, one *DFR*, one *LAR*, one *PPO*, and one *GST*, using *18S* as the endogenous control. Despite some difference being observed at one or two timepoints between qRT-PCR and RNA-seq, tested genes showed similar trends in their expression patterns as a whole, which further validated the accuracy of the transcriptome sequencing data (Figure 7B).

## 3. Discussion

### 3.1. Wound-Induced Responses of D. cochinchinensis Reveal Plants’ General Defense Mechanism against Wound Stress in Natural Conditions

By identifying the connections among different metabolic processes of *D. cochinchinensis* under wound stress, some general characteristics of plant defense mechanisms against wound stress were determined. According to our RNA-seq data, the expression of many genes in the TCA and glycolysis was upregulated from 24 h to 30 days. The plant TCA cycle provides essential precursors for respiration, amino-acid biosynthesis, and general nitrogen metabolism; moreover, it is closely involved in stress responses and cellular redox homeostasis [27]. Similarly, activated TCA cycle and glycolysis were implicated in wound responses of pumpkin [28], which indicated that these pathways might be plants’ general responses to wound stress. TCA cycle and glycolysis might function with wound stress in several ways. Firstly, under wound stress, cells around the wound may induce mitochondrial oxygen-consuming respiration, thus resulting in hypoxic conditions [29]. TCA and glycolysis could generate ATP and NADH, thus providing an emergency energy source and reducing powers under hypoxic conditions in cells for survival. Secondly, the enhanced TCA cycle, glycolysis, and glycerolipid biosynthesis could produce the obligatory component glycerol-3-phosphate that contributes to basal resistance against pathogen and induces the systemic acquired resistance in plants [30]. Thirdly, TCA cycle and glycolysis drive the downstream pathway and support the precursor for phytoalexins synthesis. In *D. cochinchinensis* stems, glycolysis could generate abundant glycose-6P, which are further converted into erythrose-4P via the upregulated *transketolase* gene. Subsequently, via a series of upregulated genes, including *3-deoxy-7-phosphoheptulonate synthase*, *3-dehydroquinate synthase*, *shikimate kinase*, *chorismate mutase*, *aspartate aminotransferase*, *glutamate/aspartate-prephenate aminotransferase*, and *arogenate/prephenate dehydratase*, erythrose-4P could be transferred into phenylalanine. Thereafter, the upregulated *phenylalanine ammonia-lyase* gene would convert phenylalanine into cinnamic acid and promote the downstream flavonoid biosynthesis (Figure 3).

Mechanical damages always harm plants with other complications such as insect feeding and pathogen invasion, especially in natural conditions. Thus, complicated responses must be activated by plants to fight against wound-caused threats. Except for the TCA cycle and glycolysis, other metabolic processes might also be involved in plants’ general defense responses to wound stress. In the present study, many genes annotated in starch and sucrose metabolism pathways were upregulated in *D. cochinchinensis* stems. Similarly, starch was consumed during the early stage of defense responses in *A. sinensis* [31], which indicated that starch and sucrose might be the important material basis of plants in responses to mechanical damage. In addition, pathways involving fatty acids were related to wound healing, insect and pathogen resistance, and signaling [32], which are in accordance with the RNA-seq data in the present study. To summarize, the TCA cycle, glycolysis, starch and sucrose metabolism, and fatty acid-involved pathways might together participate in the plants’ general defense mechanism. On the basis of the plants’ general defense mechanism, different species generate specific defense responses to survive in special growing conditions.

### 3.2. Signals Triggering the Defensive Responses in D. cochinchinensis Stems against Mechanical Damage

Mechanical damages usually occur with multiple biotic and abiotic stresses, and plants use intricate signaling systems to deal with them. A previous study indicated that several molecules, including hydrogen peroxide, jasmonic acid, salicylic acid, ethylene, and NO might participate in defense responses to varying degrees in the trunk of *D. cambodiana* and *D. cochinchinensis* against mechanical damages [33]. The RNA-seq data in this study showed that the plant hormone signal transduction pathway was upregulated at the early stage after wounding, which also suggested a complex signaling system in *D. cochinchinensis* stems under wound stress. In the plant hormone signaling pathway, some receptor genes of gibberellin, abscisic acid, ethylene, jasmonic acid, salicylic acid, and brassinosteroid were upregulated, indicating that *D. cochinchinensis* became more sensitive to these hormones under wound stress. Jasmonic acid, salicylic acid, and brassinosteroid are commonly recognized plant signals under stress [34,35]. Genes involved in the synthesis of jasmonic acid, brassinosteroid, and salicylic acid were significantly upregulated, indicating an increasing content of these hormones. Therefore, jasmonic acid, brassinosteroid, and salicylic acid might play important roles in the signaling system of *D. cochinchinensis* that deals with mechanical damages. Moreover, many genes in auxin and cytokinin transduction pathways were significantly downregulated from 3 days to 30 days, indicating that the growth processes in *D. cochinchinensis* slowed down under wound stress.

In addition to plant hormones, reactive oxygen species and cytosol Ca^2+^ content are important parts of the plant’s signaling system in defensive reactions and might also function in *D. cochinchinensis*. It was confirmed that glutamate triggered long-distance calcium-based plant defense signaling, similar to the nervous system in plants [36]. Similar molecular events were observed in this study as various unigenes (*glutathione S-transferase 4* and *gamma-glutamyltranspeptidase 3*) transferring glutathione to glutamate, and the expression of Ca^2+^ response genes (*CNGCs*, *Rboh*, *CDPK,* and *CaMOML*) was upregulated. The expression of peroxidase genes was also upregulated, which indicated an active oxygen burst around the wounding microenvironment in stems of *D. cochinchinensis*. However, the content change of these signals should be detected by different methods to further confirm their roles in defense reactions of *D. cochinchinensis* against mechanical damages.

### 3.3. Interplay between Wound Stress and Flavonoid Biosynthesis in D. cochinchinensis

**Figure 8:**
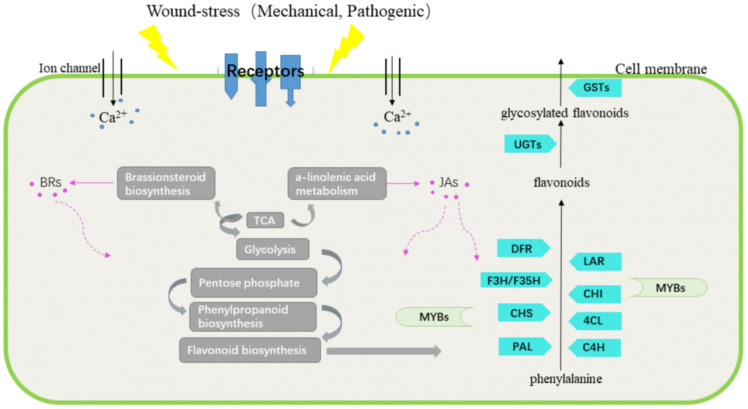
Inferred interplay of wounding and flavonoid biosynthesis in *D. cochinchinensis*.

This study presents a comprehensive interplay between wound stress and flavonoid biosynthesis in *D. cochinchinensis* over a long timeline at both the chemical and the genetic level. Combined KEGG analysis of DEGs and DEMs showed that flavonoid biosynthesis was activated throughout the overall defensive process of *D. cochinchinensis* from the short term to long term after wounding. It could be extrapolated that wound stress might promote flavonoid biosynthesis in two ways: (1) wound stress could activate the metabolic flux from primary metabolism into the flavonoid biosynthesis pathway, as the activated metabolic flux could provide abundant substrate; (2) wound stress accompanied by microorganism invasion could stimulate the plants to generate signals (e.g., Jas), which could regulate the expression of genes involved in the flavonoid biosynthesis. Tamari et al. found that methyl jasmonate (JA-Me) may mediate the wound-induced changes in flavonoid biosynthesis in *Petunia corollas* as it could promote the expression of *CHS* and *DFR* [37]. Sun et al. indicated that *PtrCHS4* promoter could be systemically responsive to wounding stimuli [38]. Naoumkina et al. revealed that yeast elicitor and the wounding signal JA-Me could induce the accumulation of medicarpin, an isoflavonoid phytoalexin, in *Medicago truncatula* cell suspensions through different mechanisms [39]. In response, the plant generates flavonoids to cope with the wound and accompanying stress [5,40]. In *D. cochinchinensis*, 7,4-dihydroxyflavan exhibits toxicity against fungi [41]. 2,4,4-Trihydroxychalcone exhibits antibacterial activity. All these studies implied a close relationship between wound stress and flavonoid biosynthesis in plants. The inferred interplay of wound stress and flavonoid biosynthesis in *D. cochinchinensis* is shown in Figure 8.

In this study, the wound-induced flavonoid biosynthesis in *D. cochinchinensis* showed periodic features. At the chemical level, different classes of flavonoids seemed to be accumulated in different periods. Chalcones and some flavones as upstream products in the flavonoid biosynthesis pathway were mainly enriched in the short term and early middle term (within 10 days) after wounding. Flavonols and isoflavone were mostly enriched after the middle term as they need more synthetic procedures. These two types of flavonoids are well-recognized phytoprotectins that could be used by plants to fight against pathogen after they break through the plant’s constructive defense system [42]. 4,4′-Dihydroxy-2-methoxychalcone, 3,7-dihydroxy-4′-methoxyflavone, liquintigenin, diosmetin, cynaroside, quercetin, and 7-hydroxy-4′-methoxyflavane are proposed to be important metabolites in defenses against wound-caused stress as the content of these compounds was persistently increased from the short-term to long-term stages after wounding. It seems that a protracted war exists between *D. cochinchinensis* and wound stress in natural conditions, and few studies have focused on the defense responses of plants over a long timeframe. Unlike other tree-like plants, *Dracaena* species do not have cambium, and they live and grow as trees depending on a couple of thin layers of vascular cells between the outer cortex and the inner middle pith [43]. They grow very slowly, and they lack competitiveness compared to the other plants in the same environment [44]. As one of the oldest plants in the world, *Dracaena* species must have evolved special defense systems to stress. These long-term defense responses seem to be the weapon used by *D. cochinchinensis* to win the war.

It was obvious that the majority of flavonoids enriched in the long term were flavonoid glycosides. Multiple glycosylated flavonoids were observed in the long-term samples, including quercetin-7-*O*-glucoside, tricin-7-*O*-rutionside, kaempferol-3-*O*-galactoside, and kaempferol-3-*O*-7-*O*-rhamnoside. Flavonoid glycosylation may be of great importance for the defensive responses of *D. cochinchinensis* against wound stress. First, glycosylation could modify the bioactivity of these flavonoids; accordingly, the plant could modulate the toxicity of these phytoprotectin and regulate active levels of various hormones in order to respond flexibly to wound stress during times of change [45,46]. Moreover, glycosylation increases the stability and solubility of flavonoid compounds and enables access to active membrane transport systems that recognize glucosylated flavonoids [24]. Unlike other plants that could secret resin, no specialized cell, such as resin duct, was observed in the microstructure of *Dracaena* species to transport the resinous material [47]. The glucosylated flavonoids would make it easy to transport the flavonoid-based resin from the synthesis locus to the wounds. In addition, the aglycones could make the resin sticky and contribute to better coverage of the wound to build a physical barrier and prevent the subsequent expansion of injury. Moreover, studies have shown that the glycosides of flavonoids exhibit good pharmacokinetic and pharmacological properties [48,49]. The multiple glycosylation of flavonoids that occurs in the long-term samples may somehow explain why the dragon’s blood generated in the prolonged accumulation process after wounding has higher medicinal value.

### 3.4. Decoding the Flavonoid Biosynthesis Pathway in D. cochinchinensis Is the Key to Effectively Producing High-Quality Dragon’s Blood

The resin, also known as dragon’s blood, produced by *D. cochinchinensis* under wound stress, is a famous traditional medicine that has been used in a wide range of clinical applications [50]. It is a plant defense outcome that simultaneously exhibits high medicinal value for humans, which makes *D. cochinchinensis* a typical inducible medicinal plant. The item inducible medicinal plant, initially proposed by Prof. Wei, refers to a plant that cannot or limitedly generates medicinal components under natural health conditions and needs to be induced to produce and accumulate abundant defensive substances having medicinal value [51]. These plants include but are not limited to *A. sinensis*, *Dalbergia odorifera*, and some *Dracaena* spp. [52]. As a typical inducible medicinal plant, *D. cochinchinensis* produces flavonoid compounds with multiple bioactive activities under wound stress. Loureirin B is a marker compound for the quality assessment of dragon’s blood [53]. According to the flavonoid biosynthesis profile of *D. cochinchinensis*, loureirin A, C, and D were produced in the early metabolism stages after wounding and stopped increasing in the long-term reactions, whereas loureirin B production started during the middle metabolism stage after wounding and accumulated at a very low speed over 17 months, which may explain the phenomenon that only trees damaged for a certain time can generate high-quality dragon’s blood. Thus, elucidating the synthesis mechanism of loureirin B is the key to the efficient induction of high-quality dragon’s blood.

Several studies have focused on the biosynthesis mechanism of flavonoids in *Draceana* spp. Zhu et al. identified 20 genes associated with flavonoid biosynthesis, 27 genes associated with flavonoid modification, and 68 genes associated with flavonoid transport on the basis of the RNA-seq of *D. cambodiana* with a chemical inducer treatment [21,22]. It was observed that four *4CL* genes, three *CHS*, two *CHI*, five *DFR*, one *LAR,* and one *F3H* were significantly upregulated after 3 days with the chemical inducer treatment. Zhang et al. reported 53 genes encoding 14 key enzymes in *D. cochinchinensis*, namely, four *PAL*, three *C4H*, nine *4CL*, three *HCT*, five *OMT*, seven *CHS*, five *CHI*, two *F3H*, three *FLS*, two *F3′5′H*, six *DFR*, one *ANS*, one *LAR*, and one *ANR*. They found that 13 DEGs were enriched after wounding stress, namely, three *PAL*, one *C4H*, two *4CL*, three *HCT*, five *CHS*, two *OMT*, one *CHI*, five *DFR*, two *F3′5′H*, three *FLS*, and one each of *F3H*, *LAR*, and *ANS* [15]. Similar to our findings, different genes were activated at different timepoints after wounding, and many genes were upregulated after 10 days. Furthermore, they reported 85 *MYBs* and 93 *bHLH* with no *WD40*. Benefiting from the reference genome sequence [54] and the updated annotation tools [25,26], we obtained the most comprehensive profile of flavonoid biosynthesis-related genes in *Dracaena* species, including 149 genes encoding 18 enzymes and 119 MYBs. To further focus on the key genes determining the quality of dragon’s blood, correlation analysis was performed on the basis of the loureirin B and C content and DEG expression. It was found that loureirin B and C followed similar kinetics with that of the expression of a group of 21 genes (Figure 7). Further studies on the function and regulation of these 21 genes would enable a better understanding of the formation mechanism of high-quality dragon’s blood.

## 4. Materials and Methods

### 4.1. Materials

Approximately 10 year old *D. cochinchinensis* plants growing in the Yunnan Branch of Institute of Medicinal Development were used in this study. The species was identified by Prof. Zhonglian Zhang. Materials were collected from trees in similar growing status and health conditions. A 3 cm long, 2 cm wide, 1 cm deep incision was cut in the trunk to cause wound stress using an alcohol-sterilized knife. The interval between incisions was >5 cm to avoid an interaction effect. Wounds were made 17 months before the sample collection date, which were collected at the same time as 0 h samples to avoid the influence by environmental changes within the time period. Samples in three biological repetitions were collected at each timepoint after wounding (0 h, 6 h, 12 h, 1 day, 3 days, 5 days, 10 days, 30 days, 2 months, 3 months, 6 months, and 17 months) (Table S1) and then were immediately frozen in liquid nitrogen and stored at −80 °C in preparation for RNA and secondary metabolite extraction. Voucher specimens of *D. cochinchinensis* were stored in the herbarium of Yunnan Branch of the Institute of Medicinal Development.

### 4.2. Methods

#### 4.2.1. Secondary Metabolites Extraction

The lyophilized vacuum samples were powdered by a mixer mill (MM 400, Retsch, Haan, Germany) at 30 Hz for 1.5 min. Then, 100 mg of freeze-dried powders were dissolved in 1.2 mL of 70% methanol solution, and vortexed for 30 s every 30 min. After 180 min, the sample was placed at 4 °C. the sample was centrifuged at 12,000 rpm for 10 min, and then the extracts were filtrated (SCAA-104, 0.22 μm pore size; ANPEL, Shanghai, China).

#### 4.2.2. UPLC Conditions

Filtrated extracts were detected using a UPLC–ESI-MS/MS system (UPLC, SHIMADZU Nexera X2, Kyoto, Japan; MS, Applied Biosystems 4500 Q TRAP, ThermoFisher, Waltham, MA, USA) with an Agilent SB-C18 (1.8 µm, 2.1 mm × 100 mm) column using a mobile phase consisting of solvent A (0.1% formic acid in pure water) and solvent B (0.1% formic acid in acetonitrile). Sample detection was conducted with a gradient program as follows: 95% A and 5% B for the starting condition; within 9 min, a linear gradient to 5% A and 95% B was programmed and then kept for 1 min; then, a composition of 95% A and 5% B was checked within 1.1 min and kept for 2.9 min. The flow velocity was set to 0.35 mL·min^−1^. The column oven temperature was set to 40 °C. The injection volume was 4 μL. The effluent was alternatively connected to an ESI triple-quadrupole linear ion trap (Q TRAP) MS.

#### 4.2.3. ESI-Q TRAP-MS/MS

The LIT and triple-quadrupole scans were obtained on a triple-quadrupole linear ion trap mass spectrometer (Q TRAP), AB4500 Q TRAP UPLC/MS/MS System, equipped with an ESI Turbo Ion-Spray interface under the operation of the software Analyst 1.6.3. After DP and CE optimization, a specific set of MRM transitions was monitored for each period according to the metabolites eluted.

#### 4.2.4. Secondary Metabolome Data Analysis

Unsupervised PCA was conducted using the statistics function within R. The HCA results of samples and metabolites were visualized by a heatmap using R. PCCs were calculated by R and presented as heatmaps. Obviously differential metabolites were selected according to VIP ≥1 and log_2_fc (fold change) ≥1. VIP values were extracted from OPLS-DA results generated using R package MetaboAnalystR. A permutation test (200 permutations) was conducted to avoid overfitting. Identified metabolites were annotated using the KEGG Compound database. (http://www.kegg.jp/kegg/compound/ (accessed on 16 September 2020)). and then mapped to the KEGG Pathway database (http://www.kegg.jp/kegg/pathway.html (accessed on 16 September 2020)). Pathways with significantly regulated metabolites were fed into MSEA (metabolite set enrichment analysis), and the significance was determined using the hypergeometric test’s *p*-values.

#### 4.2.5. RNA Extraction and Sequencing

Total RNAs from samples at different timepoints were extracted with the Trizol reagent (Invitrogen, Waltham, MA, USA). RNA concentrations were confirmed by Qubit 2.0 Flurometer (Life Technologies, Waltham, MA, USA) with a QubitVR RNA Assay Kit. After the evaluation of RNA integrity by the RNA Nano 6000 Assay Kit of the Bioanalyzer 2100 (Agilent Technologies, Palo Alto, CA, USA), total RNA was purified with poly-T oligo-attached magnetic beads. Reverse transcription of the first-strand cDNA was performed with random hexamer primer and M-MuLV Reverse Transcriptase, and then RNaseH was used to degrade the template RNA. Then, the second-strand cDNA was synthesized using DNA polymerase I and dNTP. After adenylation of the 3′ ends of DNA fragments, the adaptor with a hairpin loop structure was ligated. Specifically, 370–420 bp cDNA fragments were preferentially selected and purified with AMPure XP system (Beckman Coulter, Beverly, CA, USA). Phusion High-Fidelity DNA polymerase, universal PCR primers, and Index (X) Primer were used for PCR amplification. Subsequently, the PCR products were purified (AMPure XP system), and library quality was assessed on the Agilent Bioanalyzer 2100 system (Agilent Technologies, CA, USA). Finally, the prepared cDNA was sequenced using the Illumina Novaseq system (Illumina, San Diego, CA, USA).

#### 4.2.6. Transcriptome Data Analysis

Reads with adapter sequences, N bases, and low quality were removed using cutadapt 1.9, fqtrim 0.94, and FastQC 0.10.1 to obtain clean reads. The Q20, Q30, and GC content were calculated to ensure high quality. The genome sequence data of *D. cochinchinensis* were obtained as reference genome sequence from our previous study [54]. The alignment of the paired-end clean reads with the reference genome sequence was conducted by software Hisat2 v2.0.5. Differential expression analysis was performed using the DESeq2 R package (1.20.0). The *p*-values were calculated using Benjamini and Hochberg’s method to control the false discovery rate. Gene Ontology enrichment analysis was conducted using the cluster Profiler R package. GO terms with a corrected *p*-value <0.05 were considered to be significantly enriched. Furthermore, CDS sequences were specially annotated using the Knowledge-based Identification of Pathway Enzymes (KIPEs) method [25] and MYBs annotator [26]. Data visualization was performed using the OmicStudio tools at https://www.omicstudio.cn (accessed on 8 October 2021).

#### 4.2.7. Combined Analysis of Metabolome and Transcriptome

The EXCEL file containing both metabolism and transcriptome analysis results was used to calculate the Pearson correlation coefficients according to the fold changes of DEGs and each DEM. Correlations corresponding to a coefficient with *R*^2^ > 0.8 were selected. DEGs and DEMs annotated in specific KEGG pathways with high correlation scores were selected to draw the correlation network.

#### 4.2.8. qRT-PCR Validation for Putative Genes Related to Flavonoid Biosynthesis

The qRT-PCR was performed using a LightCycle^®^ 96 real-time OCR system (Roche, Switzerland). Reactions were prepared in a total volume of 10 μL containing 5 μL of Transtart^®^ TOP Green qPCR SuperMix (Transgen, Beijing, China), 0.5 μL of cDNA, 3.5 μL of RNase Free Water, and 0.5 μL each of the forward and reverse primers. The PCR program was 95 °C for 10 min, followed by 45 cycles of 95 °C for 5 s, 60 °C for 15 s, and 72 °C for 20 s. The melting curves were analyzed at 65–95 °C after 45 cycles. Each qRT-PCR analysis was performed in triplicate. The melting curve, melting temperature, and Ct value were output by LightCycle^®^ 96 SW1.1 Software, among which the Ct value was used to calculate the expression of reference genes in different samples. PCR analyses were implemented using *18S* sequence as endogenous control. Primers and amplicon lengths are listed in Table S5.

## 5. Conclusions

The present study investigated the systematic responses of *D. cochinchinensis* to wound stress through transcriptomics and metabolomics analyses. Metabolic processes and molecular events of *D. cochinchinensis* under wound stress both showed phase characteristics. We propose that complex responses involving the TCA cycle, glycolysis, starch and sucrose metabolism, and flavonoid biosynthesis play a role in the defense mechanism against wound stress in natural conditions. Jas, Br, abscisic acid, and gibberellin hormones may be wounding signals regulating the defensive responses. Flavonoid biosynthesis and modification are the main strategies of *D. cochinchinensis* in the long-term responses to wound stress. The comprehensive profile of flavonoid biosynthesis and its modification in *D. cochinchinensis* over time after wounding were presented in this study. On the basis of the findings in this study, further studies are recommended to be conducted in the following aspects: (1) to verify the function of genes related to the dragon’s blood formation discovered in this study according to a stable genetic transformation system; (2) to determine the main wounding signaling molecules and signal transduction pathways which dominate the wound-induced resin formation process through exogenous treatment. The former would help to figure out the biosynthesis pathway of main active substances of dragon’s blood. The latter would help to reveal an efficient elicitor for the development of an artificial induction method of dragon trees.

## Figures and Tables

**Table 1:** Enriched KEGG pathways according to co-analysis of DEGs and DEM.

Pathway	Enriched Number of DEGs/DEMs						Gene Symbol	Compound
	1 Day	3 Days	5 Days	10 Days	30 Days	17 Months		
Phenylalanine metabolism	15/1	8/1	10/1	15/1	8/1	4/1	*GOT1*, *hisC*, *PAL*, *AOC3*, *HPD*	Cinnamic acid, salicylic acid
Ubiquinone and other terpenoid-quinone biosynthesis	21/2	17/3	18/3	17/3	16/3	9/3	*CYP73A*, *4CL*, *HPD*, *VTE3*, *VTE1*, *E2.1.1.95*, *HPT*	Cinnamic acid, *p*-coumaric acid, 4-hydroxybenzoic acid, 3-[(1-carboxyvinyl)oxy]benzoic acid
Stilbenoid, diarylheptanoid, and gingerol biosynthesis	7/2	7/3	8/4	7/4	6/4	5/4	*CYP73A*, *HCT*, *CYP98A*, *E2.1.1.104*	Resveratrol, 5-*O*-*p*-coumaroylquinic acid, chlorogenic acid, piceatannol
Aminoacyl-tRNA biosynthesis	28/1	3/1	5/1	6/1	4/1	-	*EARS*, *VARS*, *gatA*, *LARS*, *AARS*, *DARS1*, *NARS*, *RARS*, *glyQ*, *PARS*, *TARS*, *HARS*, *FARSA*, *YARS*, *WARS*	10-Formyltetrahydrofuran
Isoquinoline alkaloid biosynthesis	12/1	7/2	10/1	13/1	7/1	2/1	*GOT2*, *TYDC*, *E1.10.3.1*, *AOC3*	*p*-Coumaric acid, Protocatechualdehyde
One-carbon pool by folate	10/1	6/1	7/1	13/1	8/1	-	*gcvT*, *glyA*, *MTHFS*, *MTHFD1L*, *metF*	10-Formyltetrahydrofuran
Flavonoid biosynthesis	11/4	16/12	20/18	17/19	16/19	13/17	*CYP73A*, *CHS*, *E5.5.1.6*, *CYP75B1*, *ANR*, *LAR*, *HCT*, *C3’H*, *E2.1.1.104*	7,4′-Dihydroxyflavone, pinocembrin, isoliquiritigenin, liquiritigenin, apigenin, galangin, naringenin chalcone, pinobanksin, naringenin, afzelechin, epiafzelechin, luteolin, eriodictyol, aromadendrin, quercetin, dihydroquercetin, 5-*O*-*p*-coumaroylquinic acid, chlorogenic acid, naringenin-7-*O*-glucoside, neohesperidin, butin
Tyrosine metabolism	18/1	12/2	17/1	26/4	16/4	3/4	*FAH*, *maiA*, *HPD*, *ADH1_7*, *AOC3*, *GOT1*, *TDC-1*, *E1.10.3.1*	2,5-Dihydroxybenzaldehyde, tyrosol,2,5-dihydroxybenzoic acid, gentisic acid, homogentisic acid, *p*-coumaric acid
Flavone and flavonol biosynthesis	2/1	3/4	5/7	5/9	4/8	3/8	*CYP75B1*, *E2.1.1.76*	Apigenin, acacetin, luteolin, quercetin, 3,7-di-*O*-methylquercetin, laricitrin, cosmosiin, cynaroside, astragalin
Phenylpropanoid biosynthesis	46/5	36/9	57/7	61/9	40/8	24/6	*PAL*, *CYP73A*, *F5H*, *4CL*, *CSE*, *HCT*, *C3’H*, *CYP73A*, *E2.1.1.104*, *REF1*, *CCR*, *CAD*, *E1.11.1.7*	Cinnamic acid, *p*-coumaryl alcohol, *p*-coumaric acid, caffeic acid, coniferyl alcohol, 5-*O*-*p*-coumaroylquinic acid, coniferin, chlorogenic acid, 1-*O*-Sinapoyl-d-glucose, scopolin, ferulic acid
Glycine, serine, and threonine metabolism	-	17/1	21/1	33/1	15/1	3/1	*PGAM*, *gpmI*, *hprA*, *HPR2_3*, *serA*, *serB*, *SDS*, *trpA*, *glyA*, *betB*, *PIPOX*, *GGAT*, *AGXT2*, *gcvT, GCSH*, *DLD*, *GLDC*, *ltaE*, *AOC3, SDS*	Betaine
Arginine and proline metabolism	-	10/1	14/2	25/2	15/1	*-*	*proB*, *proA*, *PRODH*, *P4HA*, *E4.1.1.19*, *aguA*, *GOT1*, *speD*, *PAO4*, *ALDH*, *speE*, *MPAO*, *SMOX*	*p*-Coumaroylputrescine, *N*-feruloylputrescine

## Data Availability

The RNA-seq data were deposited in the Genome Sequence Archive (Genomics, Proteomics, and Bioinformatics 2021) in the National Genomics Data Center (Nucleic Acids Res 2022), and in the China National Center for Bioinformation/Beijing Institute of Genomics, Chinese Academy of Sciences (GSA: CRA006271), which are publicly accessible at http//:ngdc.cncb.ac.cn/search/?dbId=gsa&q=cra006271 (accessed on 1 March 2022).

## Supplementary Materials

The following supporting information can be downloaded at https://www.mdpi.com/article/10.3390/molecules27144514/s1, Figure S1. Metabolic profile of samples at different timepoints after wounding; Figure S2. General information of DEGs at different wounding timepoints; Figure S3. KEGG enrichment of both DEGs and DEMs; Table S1. Sample information; Table S2. Continuously increased flavonoids in *D. cochinchinensis* after wounding; Table S3. General information of transcriptome sequencing; Table S4. Information of upregulated genes presented in Figure 3; Table S5. Primers used for qRT-PCR. Additional File1: Information and content of metabolites. Additional File2: Gene annotation and expression record. Additional File3: Flavonoid compounds enriched at different timepoints after wounding. Additional File4: Genes involved in flavonoid biosynthesis in *D. cochinchinensis*.

## Sample Availability

Samples of the trees are available from the authors.
