# Supplementary material for: Transcriptomics and Metabolomics Analyses Reveal Defensive Responses and Flavonoid Biosynthesis of Dracaena cochinchinensis (Lour.) S. C. Chen under Wound Stress in Natural Conditions
Source: Molecules. 2022 Jul 15;27(14):4514. doi: 10.3390/molecules27144514 (PMC9320494; doi:10.3390/molecules27144514)
Supplement: Supplementary file 1 [file molecules-27-04514-s001.zip › supplementary materials.pdf]

Table S1 Samples information

**Sample No**	**Group No**	**Wounding Time**	**Analysis method**
W_0h-1/ L_40-0h	W_0h	No wounding	Transcriptome &
W_0h-2/ L_54-0h			Metabolome
W_0h-3/ L_66-0h			
L_40-6h	W_6h	6 hours	Transcriptome
L_54-6h			
L_66-6h			
L_40-12h	W_12h	12 hours	Transcriptome
L_54-12h			
L_66-12h			
W_1d-1/ L_40-24h	W_1d/W_24h	24 hours	Transcriptome &
W_1d-2/ L_54-24h			Metabolome
W_1d-3/ L_66-24h			
W_3d-1/ L_40-3d	W_3d	3 days	Transcriptome &
W_3d-2/ L_54-3d			Metabolome
W_3d-3/ L_66-3d			
W_5d-1/ L_40-5d	W_5d	5 days	Transcriptome &
W_5d-2/ L_54-5d			Metabolome
W_5d-3/ L_66-5d			
W_10d-1/ L_40-10d	W_10d	10 days	Transcriptome &
W_10d-2/ L_54-10d			Metabolome
W_10d-3/ L_66-10d			
W_30d-1/ L_40-30d	W_30d	30 days	Transcriptome &
W_30d-2/ L_54-30d			Metabolome
W_30d-3/ L_66-30d			
W_2m-1	W_2m	2 months	Metabolome
W_2m-2			
W_2m-3			
W_3m-1	W_3m	3 months	Metabolome
W_3m-2			
W_3m-3			
W_6m-1	W_6m	6 months	Metabolome
W_6m-2			
W_6m-3			
W_17m-1	W_17m	17 months	Transcriptome &
W_17m-2			Metabolome
W_17m-3			

Table S2 Continuously Increased Flavonoids in *D. cochinchinensis* after wounding

Compound Name	Classification	Relative Content									
		0d	1d	3d	5d	10d	30d	2m	3m	6m	17m
7-hydroxyflavone	Flavonoid	9	9	9	9	36435.67	115581.3	181513.3	223640	1366600	568170
7,4-dihydroxyflavan	Other Flavonoids	950.4	1876.467	2565.567	23745.33	442120	1396800	1629233	3027667	2016833	871066.7
6,7-Dihydroxyflavone	Flavonoid	12341.8	15271.5	11379.23	34493.33	3832000	15307667	17182667	22240667	35125000	29431333
Daidzein	Isoflavones	9	9	9	9	231000	852960	961773.3	1328800	2020167	1698767
7,4'-Dihydroxyflavone	Flavonoid	11570.17	21879.67	14564.6	47980.33	4950933	23789333	21841667	36495000	51134333	44575000
Pinocembrin (Dihydrochrysin)	Dihydroflavone	9	9	16503	1035103	1395233	837726.7	1818267	1262637	2640733	915556.7
Isoliquiritigenin	Chalcones	28400.67	20620.67	86390	2756300	41503333	45859667	43133333	45361333	37405333	19816333
2,4,4'-trihydroxychalcone	Flavonoid	27102	23857	89847	2732300	40357333	45012667	41884333	43996667	36329000	19424000
Liquiritigenin	Dihydroflavone	2503.067	3411.8	9910.5	218013.3	2746167	3166233	5045567	5517667	12444667	5741833
7-hydroxy-4'-methoxyflavane	Flavonoid	1910.2	3795.467	2114.2	6970.4	213590	822013.3	394956.7	295990	1611633	1212633
2, 4, 4'-trihydroxydihydrochalcone	Chalcones	9	558.7667	9	9082.7	807850	1508733	1790133	2521433	525930	191420
7-Hydroxy-3-(4-hydroxybenzylidene)chroman-4-one	Flavonoid	9	12308.33	8833.067	9271.633	19795.67	37722.67	43689	53077	137430	161280
Apigenin	Flavonoid	9	9	9	36723	674533.3	1709400	2737067	2746500	2278367	1763767
Galangin (3,5,7-Trihydroxyflavone)	Flavonoid	9	9	9	46440.33	701863.3	1981867	3078633	4170467	2464400	1488000
Echinatin	Chalcones	7774.367	9543.133	6923.3	31201.33	2991733	13319000	13837333	18510667	22551667	19102000

2, 4-dihydroxy-4'-methoxychalcone	Chalcones	9	9	9	10116.37	560666.7	657273.3	1298467	767566.7	1313667	976586.7
4, 4'-dihydroxy-2-methoxychalcone	Chalcones	2574.733	5288.867	3929.567	34095	756030	2621167	2049533	1420700	8663700	5679733
4,4'-Dihydroxy-2'-methoxychalcone (3-Deoxysappanchalcone)	Chalcones	9	9	9	9	6704.2	7116.833	12403.1	16938.87	41753.67	37021
2', 4'- dihydroxy-4-methoxydihydrochalcone	Chalcones	16964	21565.33	17830.33	95881.67	3554567	11856667	12206000	16570667	15831667	10632333
Loureirin C	Chalcones	9	2983.567	2548.733	27428	2135600	4435867	12267667	13622667	19592667	4096233
Epiafzelechin	Flavanols	9	9	9	9	9	16583.67	9	25309	86350.67	72092.67
Genkwanin (Apigenin 7-methyl ether)	Flavonoid	550.4	9	9	9	12219.4	13415	46363.33	25399.33	43986	40437
3′, 7-dihydroxy-4′-methoxyflavone	Flavonoid	7139.467	14208.67	6713.833	33805	425236.7	874493.3	435620	117933.7	6200233	4397100
Acacetin	Flavonoid	9	9	9	9	11720.07	9775.8	48113.33	27553.33	48139.67	42236.33
Prunetin (5,4'-Dihydroxy-7-methoxyisoflavone)	Isoflavones	9	9	9	2041.033	37403.33	44836.67	134223.3	103813	151780	141443.3
Calycosin	Isoflavones	9604	11532.1	5547.133	39720	425090	796213.3	438450	139434.3	6507233	4372933
Luteolin (5,7,3',4'-Tetrahydroxyflavone)	Flavonoid	9	9	5901.1	32817.33	104195.3	51265	148936.7	72055	100840.7	48549.33
Loureirin A	Chalcones	1900.367	8820.7	31165	681273.3	3358100	2978300	4451133	1824067	2437267	1495233
Cochinchinenin A (4'-Hydroxy-2,6-dimethoxydihydrochalcone)	Chalcones	47273.67	67151	78089.33	86502.67	1980600	5731633	8384733	9541800	16206333	12234667
Eriodictyol (5,7,3',4'-Tetrahydroxyflavanone)	Dihydroflavone	9	3932.733	32992	360150	9841900	23491000	22859667	32343333	2852767	1820400

Loureirin D	Chalcones	9	9	423.8667	37276.33	1181100	2963867	2779733	4265667	319280	205233.3
Diosmetin (5,7,3'-Trihydroxy-4'-methoxyflavone)	Flavonoid	9	9	24310.67	186683.3	602880	1035250	330153.3	728296.7	2721267	2127200
3-Methylkaempferol	Flavonols	9	9	9	1746.733	61788.33	166440	233803.3	63705.33	701076.7	722443.3
Naringenin-4',7-dimethyl ether	Flavonoid	595.5833	1205.153	1568.127	5155.2	237533.3	450353.3	1270113	700746.7	500203.3	244096.7
Quercetin	Flavonols	9	9	9	466380	6160833	13359667	20052667	13112000	36924333	31923333
7-O-Methyleriodictyol	Flavanols	13440.2	11140.83	32295.33	113017.3	6057067	22978000	42407333	42676667	30177333	12267333
2',7-Dihydroxy-3',4'-dimethoxyisoflavan	Isoflavones	101863	56422.67	320428.3	64823.33	164860	1371383	614460	266543.3	10608900	8155000
4, 4'- dihydroxy-2, 6-dimethoxydihydrochalcone	Chalcones	4381.8	6393.9	3104.1	24946.03	1176793	5708033	5930867	5966133	5860833	5937600
Loureirin B	Chalcones	24024.67	35144	60069.33	64017.67	1728400	4719467	6138300	5993767	13192000	12609333
4'- hydroxy- 2, 4, 6-trimethoxydihydrochalcone	Chalcones	40349.67	96357.67	42977.33	187352	7272400	22717333	13293667	10115700	40771333	33253667
Monohydroxy-trimethoxyflavone	Flavonoid	8578.3	15470.97	11737.73	11928.57	621886.7	636900	593203.3	62361	958330	924696.7
3-Hydroxy-5,7,8-trimethoxyflavone	Flavonoid	9111.733	15714.17	11992.3	11193.23	614763.3	623973.3	582970	63930.33	955473.3	943753.3
3,3',5-Trihydroxy-4',7-dimethoxyflavanone	Dihydroflavone	9	9	9	9	873263.3	1581687	818406.7	244650	8475000	8674033
Daidzein-7-O-glucoside(Daidzin)	Isoflavones	9	9816.667	14520.67	3746.333	446433.3	872293.3	1259833	693580	964070	2144633
Luteolin-8-C-arabinoside	Flavonoid carbonoside	3697.3	3227.933	10396.43	9	1703.267	9	8665.433	5533.367	60075	82256.67
Apigenin-7-O-glucoside(Cosmosiin)	Flavonoid	8481.967	9141.4	9071.6	39215.67	290690	211230	303950	116870.3	568833.3	639403.3
Galangin-7-O-glucoside	Flavonoid	13918.33	37597	41644.67	88091.33	190990	351096.7	435226.7	9	1203033	986623.3

Apigenin-5-O-glucoside	Flavonoid	8143.3	23904.67	17395.8	33620.33	38248	62376	76256	9	628983.3	500713.3
Naringenin-4'-O-glucoside	Flavonoid	20682	31096.33	22613	9	331010	1166367	2077133	1610233	2103267	1354500
Naringenin-7-O-glucoside (Prunin)	Dihydroflavone	9	9	9	9	1399600	5024767	3899633	7182967	1731067	724433.3
Butin-7-O-glucoside	Flavonoid	19695	29945.67	26510.33	9	249053.3	932146.7	1848000	1495233	2138867	1463867
Eriodictyol-3'-O-glucoside	Dihydroflavone	9	9	9	4922	170816.7	305580	579400	466210	242240	269280
Dihydrokaempferol-7-O-glucoside	Flavonols	9	9	9	11372.17	169330	297253.3	645616.7	440216.7	232450	284246.7
Catechin-(7,8-bc)-4β-(3,4-dihydroxyphenyl)-dihydro-2-(3H)-one	Flavanols	9	9	9	9	9	4405.867	10127.8	23016	182486.7	187130
Luteolin-7-O-glucuronide	Flavonoid	225610	185276.7	188803.3	185363.7	147940	202420	238786.7	101892	383856.7	505436.7
6-C-MethylKaempferol-3-glucoside	Flavonoid	9	9	19948.67	114465.7	352416.7	229380	487303.3	139799	321653.3	105833.3
Quercetin-3-O-galactoside (Hyperin)	Flavonols	1306.467	2562.9	2953.533	3439.433	18987.33	17837	19809	3361.8	20158.67	13998.67
Tricin-7-O-Glucoside	Flavonoid	15978.87	14194.13	20403.73	10273.67	52194.33	71264.67	51453.67	28724.33	66145.67	62691
Daidzein-7-O-apiosyl(1→6)glucoside	Flavonoid	3843.233	21678.87	6575.6	5704.667	49869.33	32986.67	58769.67	28642.67	403386.7	757023.3
Kaempferol-3-O-rutinoside(Nicotiflorin)	Flavonoid	3827.767	2159.833	3842.367	6343.133	2347.033	5746	9	9	100596	68270.67
Kaempferol-3-O-neohesperidoside	Flavonols	54338.07	44763.67	44631.37	41206.23	34800.67	57972.67	69598.33	31098.33	104206.7	120334
Kaempferol-3-O-glucoside-7-O-rhamnoside	Flavonols	61686.97	45519.67	46111.63	40786.83	56045.33	45732.67	66368	26411.33	117840	119609.3

Kaempferol-3-O-rhamnosyl(1→2)glucoside	Flavonoid	9	9	9	9	9	16403.4	8125.333	1854.067	135743.3	113223.3
Luteolin-7-O-neohesperidoside (Lonicerin)	Flavonoid	9	9	9	9	9	1911.6	2512.133	1118.933	25686.33	23630.33
Kaempferol-3-O-galactoside-4'-O-glucoside	Flavonoid	23784.47	55934.33	40319	39693.67	189123.3	173646.7	91407	125190	62020	53327.67
Hesperetin-7-O-neohesperidoside(Neohesperidin)	Dihydroflavone	9	739.7	9	9	5067.967	14065.33	9	9	191010	132333.3
Hesperetin-7-O-rutinoside (Hesperidin)	Dihydroflavone	794.4333	9	9	9	6029.2	9	9	9	146956.7	128723.3
Chrysoeriol-5,7-di-O-glucoside	Flavonoid	2692.167	2480.033	6355.333	12693.33	19760	19944.67	39037.33	8322.833	39293	49720
Isorhamnetin-3-O-neohesperidoside	Flavonols	1858.1	4930.933	3918.867	7114.4	8953.3	5885.333	5382.333	9	52261	99552.33
Chrysoeriol-6-C-glucoside-4'-O-glucoside	Flavonoid	46335.33	88422	92673.67	93135.33	218923.3	188146.7	195988.3	51892	412910	236150
Quercetin-3-O-(2''-O-galactosyl)glucoside	Flavonols	19960	24395.33	15095.67	17971.33	18963.67	24008.67	33956.67	14269.33	111577.7	95031
Chrysoeriol-7-O-(6''-feruloyl)glucoside	Flavonoid	9	9	9	9	17447.5	25203.97	48885.33	19138.33	41239.33	38202
Tricin-7-O-rutinoside	Flavonoid	19236.03	7706.233	25201.83	10357.6	201750	455876.7	186621.3	42832.33	1253447	1388467
Tricin-7-O-neohesperidoside	Flavonoid	17043.57	6114.533	24799.9	13310	190782.7	446666.7	178494.3	48201	1278357	1327533
Tricin-4'-O-(syringyl alcohol)ether-7-O-glucoside	Flavonoid	9	9	1002.267	9	612.9333	5697.3	3546.167	3427.933	316140	391150
Kaempferol-3-O-robinoside-7-O-rhamnoside (Robinin)	Flavonols	9	9	9	9	9	9	9	9	41311.33	29165.67

Naringenin-7-O-Rutinoside-4'-O-glucoside	Flavonoid	36204.33	48859.67	47083.33	65552.67	128023.3	259873.3	159163.3	120090	222996.7	183696.7

Table S3 General information of transcriptome sequencing

Sample No	Total reads	Total mapped	Error rate (%)	Q20 (%)	Q30 (%)	GC content (%)
L40_0h	41643172	93.56%	0.02	98.08	93.27	46.94
L54_0h	45448468	94.85%	0.02	98.04	94.39	46.54
L66_0h	46006786	93.65%	0.03	97.84	93.85	47.14
L40_6h	40697744	93.89%	0.03	97.88	94.03	48.93
L54_6h	41142040	93.95%	0.03	97.85	93.97	48.73
L66_6h	46403904	92.70%	0.03	97.84	94.30	47.14
L40_12h	44239526	88.77%	0.02	98.12	94.55	47.57
L54_12h	47524144	94.41%	0.03	97.82	93.85	48.38
L66_12h	45889950	93.68%	0.03	97.87	93.98	48.77
L40_24h	41816908	94.40%	0.03	97.82	93.83	47.81
L54_24h	46131730	94.17%	0.03	97.57	93.22	48.24
L66_24h	57906136	94.70%	0.03	97.94	94.09	47.82
L40_3d	43652812	94.64%	0.03	97.94	94.08	48.21
L54_3d	47634554	91.00%	0.03	96.6	91.08	49.27
L66_3d	45918918	93.79%	0.03	97.91	94.1	48.51
L40_5d	45719690	91.04%	0.03	95.48	89.23	48.47
L54_5d	43048470	90.73%	0.03	95.98	90.14	49.65
L66_5d	41966950	88.77%	0.03	96.01	90.26	49.91
L40_10d	43549800	92.58%	0.03	96.03	90.21	48.61
L54_10d	45068192	91.99%	0.03	96.06	90.28	48.56
L66_10d	47905984	90.36%	0.03	95.88	89.99	48.76
L40_30d	47437214	91.82%	0.03	96.03	90.26	47.75
L54_30d	43807720	91.33%	0.03	95.68	89.58	48.43
L66_30d	45284220	91.17%	0.03	95.45	89.16	48.57
L40_17M	43662898	94.23%	0.03	97.78	93.73	47.81
L54_17M	43906110	93.38%	0.03	97.48	93.07	48.63
L66_17M	40862750	93.56%	0.03	97.61	93.27	47.99

Table S4. Information of up-regulated genes presented in Figure 3.

Gene ID in Figure 5	Corresponding ID in sequencing Data	KEGG pathway	Annotation
2.3.3.1	evm.model.contig_2971.2;	TCA cycle	citrate synthase
	evm.model.Chr7.2058; evm.model.Chr5.45		
2.3.3.8	evm.model.Chr20.2812; evm.model.Chr20.2813		ATP citrate (pro-S)-lyase
	evm.model.Chr20.4759;		
	evm.model.Chr20.2864; evm.model.Chr7.832;		
	evm.model.Chr18.808		
	evm.model.contig_1193.3;		
	evm.model.Chr20.4787		
2.3.1.12	evm.model.Chr4.865; evm.model.Chr7.725		pyruvate dehydrogenase complex
	evm.model.Chr1.2509; evm.model.Chr20.5671		
1.2.4.1	evm.model.Chr1.419; evm.model.Chr17.259		pyruvate dehydrogenase E1 component alpha subunit
	evm.model.Chr19.654; evm.model.Chr16.138		
	evm.model.Chr19.675; evm.model.Chr16.716		
4.1.1.1	evm.model.Chr2.2164; evm.model.Chr20.2178		pyruvate decarboxylase
	evm.model.Chr20.2179; evm.model.Chr2.2166		
2.7.1.40	evm.model.Chr14.743; evm.model.Chr20.5630	Glycolysis	pyruvate kinase
	evm.model.Chr4.2552; evm.model.Chr2.2785		
	evm.model.Chr20.3639; evm.model.Chr8.161		
	evm.model.Chr7.1524; evm.model.Chr1.2679		
5.3.19	evm.model.Chr20.3729		glucose-6-phosphate isomerase
2.7.1.11	evm.model.Chr3.2413; evm.model.Chr7.400		
	evm.model.Chr12.629; evm.model.Chr2.2885		6-phosphofructokinase 1
	evm.model.Chr4.1616; evm.model.Chr18.49.1		

	evm.model.Chr5.253		
4.1.2.13	evm.model.Chr18.1015; evm.model.Chr1.1722		fructose-bisphosphate aldolase
	evm.model.Chr4.28; evm.model.Chr20.4125		
	evm.model.Chr20.5739		
1.2.1.12	evm.model.Chr4.41; evm.model.contig_1678.1		glyceraldehyde 3-phosphate dehydrogenase
	evm.model.Chr19.840; evm.model.Chr15.93		
	evm.model.Chr3.2335; evm.model.Chr1.1692		
2.7.23	evm.model.Chr3.531.1; evm.model.Chr20.3157		phosphoglycerate kinase
	evm.model.Chr20.3158; evm.model.Chr3.532		
	evm.model.Chr20.3159		
5.4.2.11	evm.model.contig_1146.4;		2,3-bisphosphoglycerate-dependent
	evm.model.Chr15.1232		phosphoglycerate mutase
	evm.model.Chr2.1439		
4.2.1.11	evm.model.Chr15.1068; evm.model.Chr2.631		enolase
	evm.model.Chr6.1003.; evm.model.Chr8.970.1		
	evm.model.Chr7.117		
3.2.1.26	evm.model.Chr4.1540; evm.model.Chr8.638	Starch and sucrose metabolism	fructan beta-(2,1)-fructosidase
	evm.model.Chr4.3034; evm.model.Chr20.4327		
	evm.model.Chr4.1541; evm.model.Chr7.492		
	evm.model.Chr20.4328		
2.7.1.4	evm.model.Chr15.750; evm.model.Chr20.5457		fructokinase
	evm.model.Chr16.1047; evm.model.Chr4.860		
	evm.model.Chr1.2506; evm.model.Chr1.3840		
	evm.model.Chr1.3841		
5.3.1.9	evm.model.Chr20.3729		glucose-6-phosphate isomerase
3.2.1.21	evm.model.Chr20.313; evm.model.contig_572.1		beta-glucosidase

	evm.model.contig_345.1;evm.model.contig_2787.1		
	evm.model.Chr9.414;evm.model.Chr17.1397		
	evm.model.Chr20.4584; evm.model.Chr12.283		
	evm.model.Chr20.4583; evm.model.Chr9.415		
	evm.model.Chr19.862; evm.model.Chr5.1168		
	evm.model.contig_1118.1;		
	evm.model.Chr19.775		
	evm.model.Chr2.1082		
3.1.44	Novel01168; evm.model.Chr2.322	Glycerophospholipid metabolism	phospholipase
	evm.model.Chr12.190;		
	evm.model.contig_1938.1		
2.3.1.15	evm.model.Chr7.1911; evm.model.Chr6.112		glycerol-3-phosphate acyltransferase
	evm.model.Chr11.1425; evm.model.Chr11.1567		
1.1.5.3	evm.model.Chr4.720		glycerol-3-phosphate dehydrogenase
2.3.1.51	evm.model.Chr8.762; evm.model.Chr2.1410		LPA acyltransferase
	evm.model.Chr17.416; evm.model.Chr2.1411		
	evm.model.Chr20.1294		
1.1.1.37	evm.model.Chr11.1647; evm.model.Chr5.609	Carbon fixation	malate dehydrogenase
	evm.model.Chr2.2167; evm.model.Chr8.301		
	evm.model.Chr7.1842		
1.1.1.40	evm.model.Chr12.659; evm.model.Chr1.3387		malate dehydrogenase
	evm.model.Chr2.2929; evm.model.Chr2.2167		
2.6.1.2	evm.model.Chr3.105; evm.model.Chr20.3129		glutamate--glyoxylate aminotransferase
	evm.model.Chr3.437; evm.model.Chr20.2983		
4.1.1.31	evm.model.Chr12.764		phosphoenolpyruvate carboxylase

2.2.2.1/2.2.1.1	evm.model.Chr4.2830; evm.model.Chr1.445 evm.model.Chr8.1013		transketolase
FabF	evm.model.Chr20.4530	Fatty acid biosynthesis	3-oxoacyl-[acyl-carrier-protein] synthase II
FabG	evm.model.Chr3.2621; evm.model.Chr11.380 evm.model.Chr3.2625		3-oxoacyl-[acyl-carrier protein] reductase
FabZ	evm.model.Chr20.4145		3-hydroxyacyl-[acyl-carrier-protein] dehydratase
FabI	evm.model.Chr2.1572; evm.model.Chr5.1351		enoyl-[acyl-carrier protein] reductase I
3.1.2.14	evm.model.Chr20.894; evm.model.Chr12.373 evm.model.Chr13.1278		fatty acyl-ACP thioesterase B
2.3.1.199	Novel00592; evm.model.Chr1.4302 evm.model.Chr9.806; evm.model.Chr2.1174 evm.model.Chr2.1173; evm.model.Chr1.4155 evm.model.Chr3.44; evm.model.Chr20.3011 evm.model.Chr1.4190; evm.model.Chr10.543	Fatty acid elongation	3-ketoacyl-CoA synthase
1.1.1.330	evm.model.Chr16.824; evm.model.Chr16.823 evm.model.Chr1.1731		17beta-estradiol 17-dehydrogenase
4.2.1.134	evm.model.Chr20.2432		very-long-chain (3R)-3-hydroxyacyl-CoA dehydratase
1.6.5.3	evm.model.Chr6.1021; evm.model.Chr4.2856 evm.model.Chr14.1228; evm.model.Chr10.871 evm.model.Chr4.125; evm.model.Chr4.2917 evm.model.Chr14.18; evm.model.Chr11.317 evm.model.Chr13.241; evm.model.Chr1.327 evm.model.Chr17.949; evm.model.Chr13.558 evm.model.Chr5.659; evm.model.Chr7.736 evm.model.Chr1.736; evm.model.Chr18.960	Oxidative phosphorylation	NAD(P)H-quinone oxidoreductase subunit 1

	evm.model.Chr14.210	
1.6.99.3	evm.model.Chr6.1021; evm.model.Chr4.2856 evm.model.Chr14.1228; evm.model.Chr10.871 evm.model.Chr4.125; evm.model.Chr4.2917 evm.model.Chr14.18; evm.model.Chr11.317 evm.model.Chr13.241; evm.model.Chr1.327 evm.model.Chr17.949; evm.model.Chr13.558 evm.model.Chr5.659; evm.model.Chr7.736 evm.model.Chr1.736; evm.model.Chr14.210 evm.model.Chr18.960	NADH dehydrogenase (ubiquinone) 1 alpha subcomplex subunit 9
1.3.5.1	evm.model.Chr19.814; evm.model.Chr13.16 evm.model.contig_2671.1	succinate dehydrogenase (ubiquinone) cytochrome b560 subunit
1.10.2.2	evm.model.Chr4.455; evm.model.Chr1.1801 evm.model.Chr7.1768; evm.model.Chr15.197 Novel01584;Novel01357 evm.model.Chr13.427; evm.model.Chr1.1453 evm.model.Chr19.574; evm.model.Chr3.1099 evm.model.Chr13.432;	ubiquinol-cytochrome c reductase cytochrome c1 subunit
1.9.3.1	evm.model.Chr3.2281; evm.model.Chr9.598 evm.model.Chr3.976; evm.model.Chr20.498 evm.model.Chr2.2441; evm.model.Chr12.291 evm.model.Chr20.5263; evm.model.contig_1757.5 evm.model.contig_1318.1	cytochrome c oxidase subunit 5b
3.6.3.14	evm.model.Chr1.914; evm.model.Chr4.2511 evm.model.Chr11.1218; evm.model.Chr20.5314	F-type H+/Na+-transporting ATPase subunit alpha

	evm.model.Chr20.5318; evm.model.Chr18.972		
	evm.model.Chr5.2525; evm.model.Chr10.439		
	evm.model.Chr20.648; evm.model.Chr5.344		
	evm.model.Chr2.2767; evm.model.Chr20.832		
	evm.model.Chr3.2113; evm.model.Chr5.2360		
	evm.model.contig_1850.3;		
	evm.model.Chr1.4311		
	evm.model.Chr12.335; evm.model.Chr5.2436		
	evm.model.Chr6.1182; evm.model.Chr3.1689		
	evm.model.Chr9.1501; evm.model.Chr2.252		
	evm.model.Chr5.2233; Novel01869		
	evm.model.Chr3.312; evm.model.Chr20.1167		
	evm.model.Chr14.89; evm.model.Chr20.5490		
	evm.model.Chr20.3778.1;		
	evm.model.Chr12.606		
	evm.model.Chr12.605; evm.model.Chr2.3052		
	evm.model.Chr1.3054; evm.model.Chr4.193		
	evm.model.Chr4.2126; evm.model.Chr12.479		
	evm.model.Chr20.5339		
3.6.3.6	evm.model.Chr2.352; evm.model.Chr5.2271		H+-transporting ATPase
	evm.model.Chr2.208; evm.model.Chr5.897		
	evm.model.Chr5.924		
3.6.1.1	evm.model.Chr18.891; evm.model.Chr4.253		inorganic pyrophosphatase
	evm.model.Chr20.2405; evm.model.Chr2.3438		
	evm.model.Chr3.2430		
2.3.1.50	evm.model.Chr7.256; evm.model.Chr8.825	Sphingolipid metabolism	serine palmitoyltransferase

	evm.model.Chr16.150; evm.model.Chr19.630		
1.1.1.102	evm.model.Chr1.185; evm.model.Chr1.236		3-dehydrosphinganine reductase
11413169	evm.model.Chr15.1328; evm.model.Chr15.1323		sphinganine C4-monooxygenase
	evm.model.Chr6.303; evm.model.Chr6.302		
	evm.model.Chr15.1324; evm.model.Chr15.1327		
	evm.model.Chr6.299; evm.model.Chr11.1684		
	evm.model.Chr6.301; evm.model.Chr6.305		
	evm.model.Chr6.306; evm.model.Chr9.1413		
4.1.2.27	evm.model.Chr16.49		sphinganine-1-phosphate aldolase
3.1.1.4	evm.model.Chr2.1410; evm.model.Chr3.132		LPA acyltransferase
	evm.model.Chr17.416; evm.model.Chr2.1411		
	evm.model.Chr20.2959; evm.model.Chr20.1294		
1.13.1112	evm.model.Chr9.81; evm.model.Chr6.820		lipoxygenase
	evm.model.Chr7.2037; evm.model.Chr7.2038	alpha-Linolenic acid metabolism	
4.2.1.92	evm.model.Chr9.767		hydroperoxide dehydratase
1.3.1.42	evm.model.contig_556.2;		12-oxophytodienoic acid reductase
	evm.model.Chr20.3876		
	evm.model.Chr1.3862; evm.model.contig_62.1		
	Novel02676; Novel02666; Novel02428		
	evm.model.contig_556.2;		
	evm.model.contig_62.1		
OPLC1	evm.model.Chr1.981; evm.model.Chr1.2313		OPC-8:0 CoA ligase 1
ACX	evm.model.Chr12.976; evm.model.Chr8.695		acyl-CoA oxidase
	evm.model.Chr20.2144		
2.3.1.16	evm.model.Chr12.976; evm.model.Chr8.695		acetyl-CoA acyltransferase 1
	evm.model.Chr1.1393; evm.model.Chr8.379		

2.5.1.54	evm.model.Chr15.1163; evm.model.Chr3.1952 evm.model.Chr9.634; evm.model.Chr3.1869	Phenylalanine, tyrosine and tryptophan biosynthesis	3-deoxy-7-phosphoheptulonate synthase
4.2.3.4	evm.model.Chr8.1361		3-dehydroquinate synthase
4.2.1.10/1.1.1.25	evm.model.Chr17.257		shikimate dehydrogenase
2.7.1.71	evm.model.Chr3.250; evm.model.Chr3.248		shikimate kinase
2.5.1.19	evm.model.Chr7.97		3-phosphoshikimate 1-carboxyvinyltransferase
4.2.3.5	evm.model.Chr6.1672		chorismate synthase
5.4.99.5	evm.model.Chr5.741; evm.model.Chr9.1565 evm.model.Chr2.328		chorismate mutase
2.6.1.78	evm.model.contig_1674.3; evm.model.Chr20.5265 evm.model.contig_2886.3		bifunctional aspartate aminotransferase
4.2.1.51	evm.model.Chr20.2744; evm.model.Chr20.4648 evm.model.Chr20.4418; evm.model.Chr20.5847		prephenate dehydratase
2.2.1.7	evm.model.Chr11.1630; evm.model.Chr3.2463 evm.model.Chr20.1372; evm.model.Chr20.4961	Terpenoid backbone biosynthesis	1-deoxy-D-xylulose-5-phosphate synthase
5.3.3.2	evm.model.Chr16.875; evm.model.Chr19.763		isopentenyl-diphosphate Delta-isomerase
2.5.1.10	evm.model.Chr20.63; evm.model.Chr7.742 evm.model.Chr5.2637; evm.model.Chr2.3348		farnesyl diphosphate synthase
4.6.1.12	evm.model.contig_151.4		2-C-methyl-D-erythritol 2,4-cyclodiphosphate synthase
2.7.1.148	evm.model.Chr14.358		4-diphosphocytidyl-2-C-methyl-D-erythritol kinase
2.5.1.29	evm.model.Chr20.63; evm.model.Chr7.742 evm.model.Chr5.2637		geranylgeranyl diphosphate synthase
DWF5	evm.model.Chr7.1366; evm.model.Chr5.996	Steroid biosynthesis	7-dehydrocholesterol reductase
CYP51G1	evm.model.Chr12.161		sterol 22-desaturase

STE1	evm.model.Chr18.672		Delta7-sterol 5-desaturase
HYD1	evm.model.Chr4.2642; evm.model.Chr16.249		cholestenol Delta-isomerase
SMO1	evm.model.Chr4.675; evm.model.contig_1789.1 evm.model.contig_1789.2; evm.model.Chr1.457 evm.model.Chr4.2811; evm.model.Chr4.2812		methylsterol monooxygenase 1-1
5.4.99.8	evm.model.Chr2.843		cycloartenol synthase
1.14.13.32	evm.model.Chr3.961; evm.model.Chr18.263 evm.model.Chr20.519		squalene monooxygenase
2.5.1.21	evm.model.Chr3.603; evm.model.Chr20.3205		farnesyl-diphosphate farnesyltransferase
90B/724E	evm.model.Chr13.10; evm.model.Chr3.1208.2	Brassinosteroid biosynthesis	steroid 22-alpha-hydroxylase
85A1/2	evm.model.Chr20.3521		brassinosteroid-6-oxidase 1
1.14.13.11	evm.model.Chr2.105; evm.model.Chr14.1034 evm.model.Chr20.2065	Stilbenoid, diarylheptanoid and gingerol biosynthesis	trans-cinnamate 4-monooxygenase
23.1.133	evm.model.Chr2.1867; evm.model.Chr10.1097		hydroxycinnamoyltransferase
1.14.13.36	evm.model.Chr17.363		5-O-(4-coumaroyl)-D-quinate 3'-monooxygenase
2.1.1.295	evm.model.Chr13.626; evm.model.Chr10.592 evm.model.Chr13.634; evm.model.Chr10.591 evm.model.Chr10.581	Ubiquinone and other terpenoid-quinone biosynthesis	MPBQ/MSBQ methyltransferase
2.1.1.95	evm.model.Chr1.160; evm.model.Chr1.158		tocopherol O-methyltransferase
2.3.1.74	evm.model.Chr19.559; evm.model.Chr1.1148 evm.model.Chr1.1139; Novel01759 evm.model.Chr1.1144; evm.model.Chr1.1145	Flavonoid biosynthesis	chalcone synthase
23.1.170	evm.model.Chr19.559; evm.model.Chr1.1148 evm.model.Chr1.1139; Novel01759 evm.model.Chr1.1144; evm.model.Chr1.1145		chalcone synthase
5.5.1.6	evm.model.Chr17.1319		chalcone isomerase

FLS2	evm.model.Chr6.344; evm.model.Chr7.749	Plant-pathogen interaction	LRR receptor-like serine
MKK1/2	evm.model.Chr11.1211; evm.model.Chr20.5361		mitogen-activated protein kinase kinase 1
WRKY33	evm.model.Chr20.2278;<br>evm.model.Chr14.1275.1<br>evm.model.Chr11.1090; evm.model.Chr20.2279		WRKY transcription factor 33
RIN4	evm.model.Chr1.4090; evm.model.Chr19.22		RPM1-interacting protein 4
PBS1	evm.model.Chr16.123		serine/threonine-protein kinase PBS1
CNGCs	evm.model.Chr1.3721; evm.model.Chr3.1616<br>evm.model.Chr9.142		cyclic nucleotide gated channel
CDPK	evm.model.Chr13.605; evm.model.Chr16.647<br>evm.model.Chr3.227; evm.model.Chr7.1279<br>evm.model.Chr3.307; evm.model.Chr20.3081<br>evm.model.Chr3.2714; evm.model.Chr9.391<br>evm.model.Chr2.2303; evm.model.Chr8.909<br>evm.model.Chr4.2486; evm.model.Chr20.4123<br>evm.model.Chr20.4122		calcium-dependent protein kinase
Rboh	evm.model.Chr16.267; evm.model.Chr16.266<br>evm.model.Chr2.3104		respiratory burst oxidase
CaMOML	evm.model.Chr13.636; evm.model.Chr6.1589<br>evm.model.Chr1.3569; evm.model.Chr11.296<br>evm.model.Chr8.1532; evm.model.Chr14.176<br>evm.model.Chr17.311; evm.model.Chr20.5454<br>evm.model.Chr11.514; evm.model.Chr20.1295<br>evm.model.Chr20.1291;<br>evm.model.contig_2997.4<br>Novel02697; evm.model.Chr2.2244		calcium-binding protein CML

	evm.model.Chr5.2006; evm.model.Chr5.1217		
	evm.model.Chr1.1811; evm.model.Chr11.384		
	evm.model.Chr4.2824		
PYR/PYL	evm.model.Chr12.102; evm.model.Chr4.2573	Plant hormone signal transduction	abscisic acid receptor PYR/PYL family
	evm.model.Chr1.813; evm.model.Chr18.38		
	evm.model.Chr14.1254		
SnRK2	evm.model.Chr4.1921; evm.model.Chr4.1920		serine/threonine-protein kinase SRK2
	evm.model.Chr8.748		
PP2C	evm.model.Chr5.390; evm.model.Chr10.623		protein phosphatase 2C
	evm.model.Chr4.813		
ABF	evm.model.Chr4.452		ABA responsive element binding factor
JAR1	evm.model.Chr5.364; evm.model.contig_2641.1		jasmonic acid-amino synthetase
JAZ	evm.model.Chr15.304; evm.model.Chr16.149		jasmonate ZIM domain-containing protein
	evm.model.Chr3.47; evm.model.Chr6.727		
	evm.model.Chr3.48; evm.model.Chr20.3018		
	evm.model.Chr6.726; evm.model.Chr2.1715		
	evm.model.Chr1.3225; evm.model.Chr15.254		
MYC2	evm.model.Chr1.127; evm.model.Chr8.1112		transcription factor MYC2
NPR1	evm.model.Chr13.1381; evm.model.Chr11.414		regulatory protein NPR1
	evm.model.Chr20.1286		
TGA	evm.model.Chr20.4351; evm.model.Chr7.2030		transcription factor TGA
	evm.model.Chr20.4351; evm.model.Chr7.2029		
	evm.model.Chr2.1060		
GH3	evm.model.Chr20.223; evm.model.Chr3.1556		auxin responsive GH3 gene family

Table S5 Primers used for qRT-PCR

Gene Name	Forward Primer (5′-3′)	Reverse Primer (5′-3′)
CHS_Chr19.559	CGCCCCAAGTCCCACATC	AACGTGACGGCGGTGATCT
CHS_Chr1.1139	TCGAGGGGCATCTAAGGGAA	GAGATCCCCAACGGCTTGAA
CHS_Chr1.1144	CGCAATCGAGGGGCATCTAA	TATGATTCCGGGCACATCCG
CHI_Chr11.863	TCAAGCACAATGCAATCGGG	CCATGCGAGCTGACACAAGA
DFR_Chr5.2202	GCAAGCACGGCACACATAAT	CCAGATCCCCACGGTGAAG
LAR_Chr3.2901	TCTCAGTTCTTGTGATTGGTGC	GCGGATGACGAAGGTCGAA
GST_Chr11.1030	TTGGGATTGCTGACATCGCT	AGCCCATCTCCAAACGACAG
MYB_Chr1.2784	GAACAGCCACCTGAGCAAGA	AGTAGCTGTCCGCGATGATG
MYB_Chr4.2326	CTCCATTCCCTTCTCGGCAA	TGTCCTTGTTCTTGGCCGTT
PPO_Chr15.131	GAGCAGCAGCCCCATAAGAC	AGGGCCAGAAGATTCCAACTG
OMT_Chr16.23	GGACGGAAAGATAACGGCCA	TTATCGGCGACTCCAGCTTC
OMT_Chr18.210	TGGCGAGTAAACATTGGGCT	GCCAGCTATCGAGAAGGACC
OMT_Chr6.1100	ACGGTGCCAGTGTTGAAAGA	ACGAAGGGGTTGCCGAAATA
OMT_Chr6.1113	AGGGGACGATGGATGCAATG	CCACAACAAATGGCAGGTCG
OMT_Chr6.1127	AGCGGCATCTTCTCCTCTACG	CGTGAGCGGTGATGTAGGGA
18S	GCAGATGGAGGAGGATAGGGTAG	TAAAAGCAGACTTCTCTCCCCC

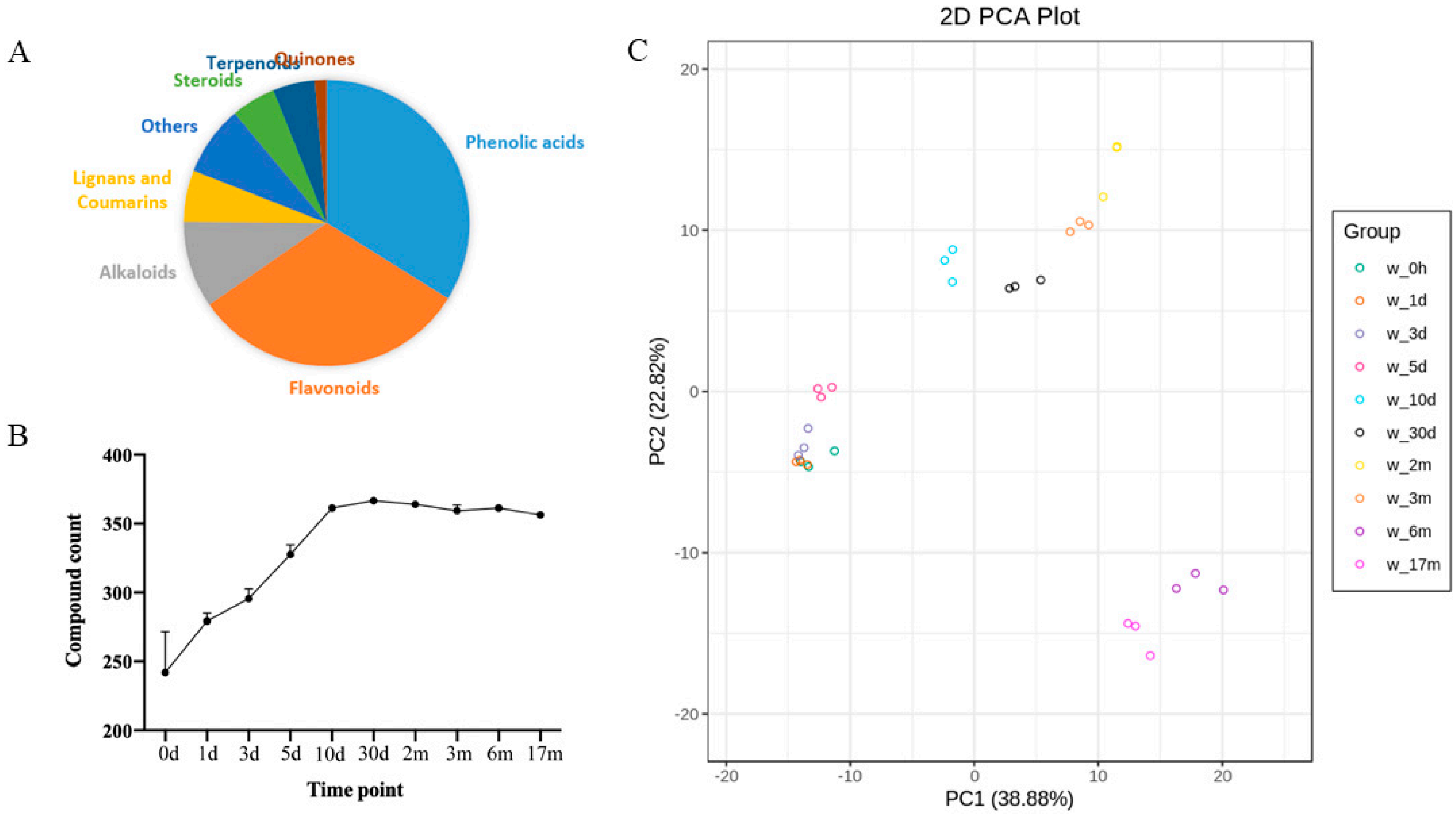

Figure S1. Metabolic profile of samples at different time after wounding

(A) Types of secondary metabolites; (B) Number change of compounds over time after wounding;

(B) PCA analysis of all the samples.

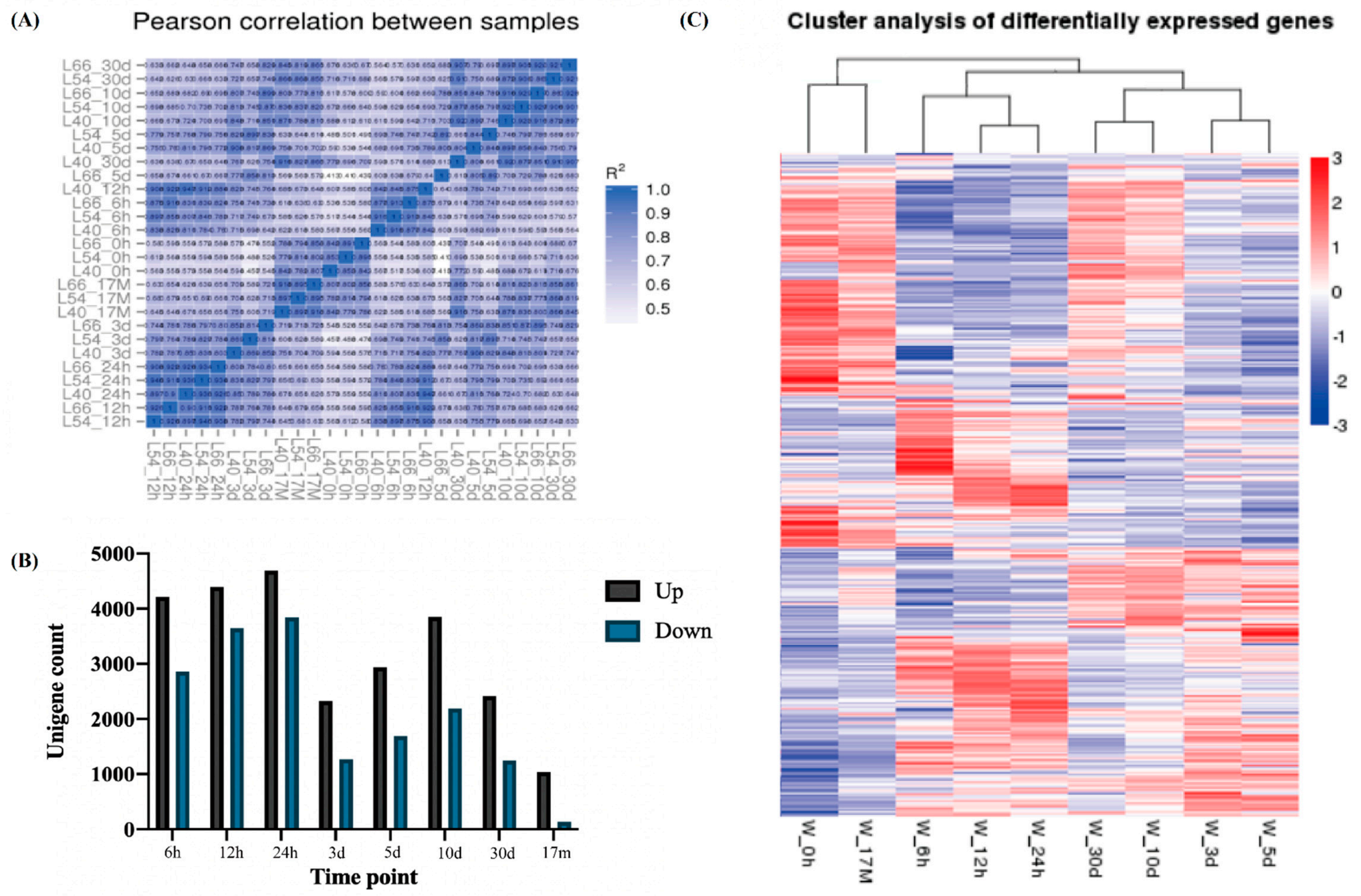

Figure S2. General information of DEGs at Different Wounding Time Points

(A) Correlation between samples; (B) Number of up-regulated and down-regulated DEGs at different time points; (C) Cluster analysis of DEGs at different time points.

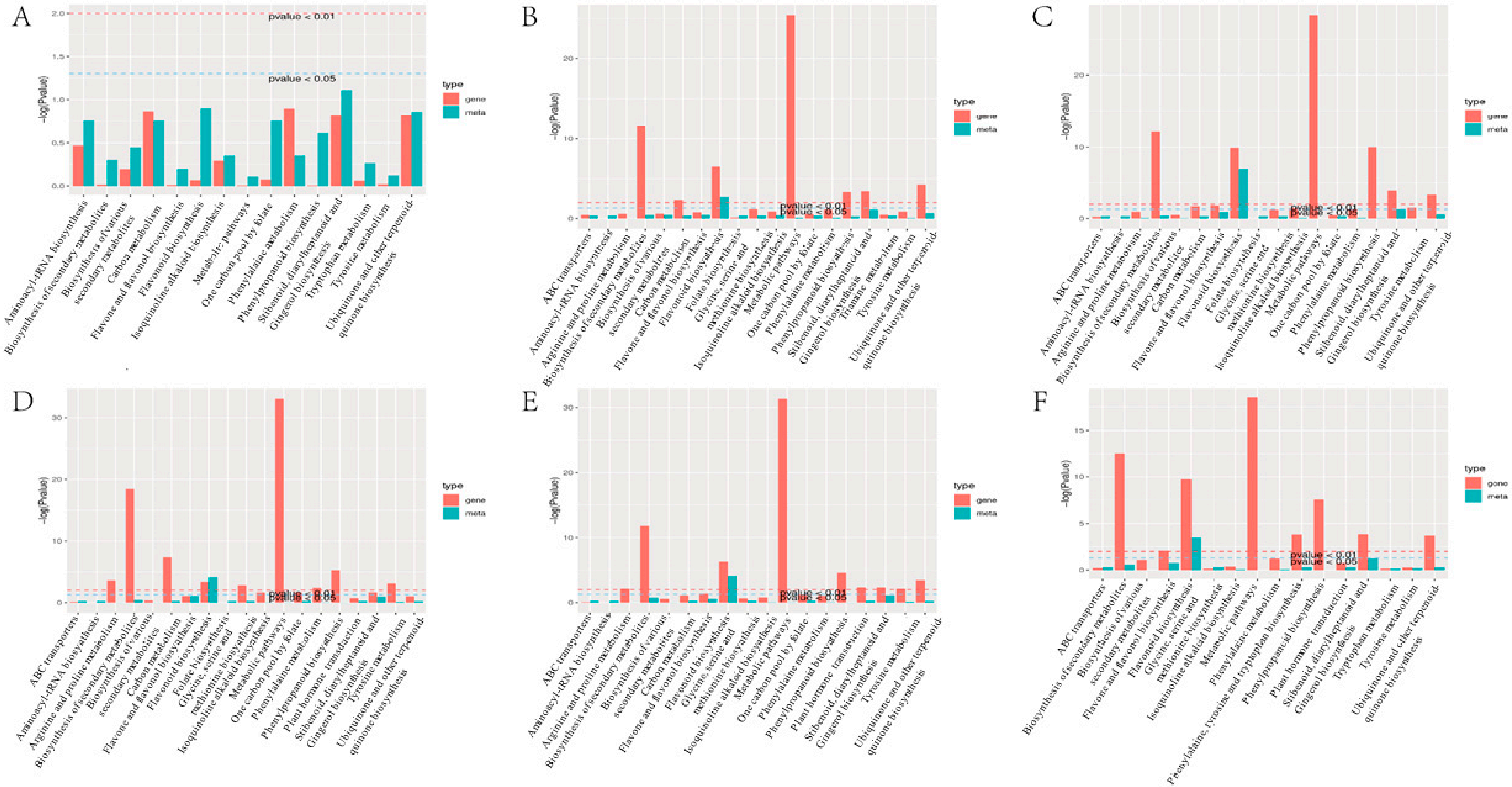

Figure S3. KEGG enrichment of both DEGs and DEMs

A:24h vs 0h; B:3d vs 0h; C:5d vs 0h; D: 10d vs 0h; E: 30d vs 0h; F: 17m vs 0h.
